# Insights into the activity of single-atom Fe-N-C catalysts for oxygen reduction reaction

**DOI:** 10.1038/s41467-022-29797-1

**Published:** 2022-04-19

**Authors:** Kang Liu, Junwei Fu, Yiyang Lin, Tao Luo, Ganghai Ni, Hongmei Li, Zhang Lin, Min Liu

**Affiliations:** 1 Hunan Joint International Research Center for Carbon Dioxide Resource Utilization, School of Physics and Electronics, Central South University, Changsha, 410083 Hunan P. R. China; 2 School of Metallurgy and Environment, Central South University, Changsha, 410083 Hunan P. R. China

**Keywords:** Chemical physics, Computational chemistry, Electrocatalysis

## Abstract

Single-atom Fe-N-C catalysts has attracted widespread attentions in the oxygen reduction reaction (ORR). However, the origin of ORR activity on Fe-N-C catalysts is still unclear, which hinder the further improvement of Fe-N-C catalysts. Herein, we provide a model to understand the ORR activity of Fe-N_4_ site from the spatial structure and energy level of the frontier orbitals by density functional theory calculations. Taking the regulation of divacancy defects on Fe-N_4_ site ORR activity as examples, we demonstrate that the hybridization between Fe 3*dz*^2^, 3*dyz* (3*dxz*) and O_2_ π* orbitals is the origin of Fe-N_4_ ORR activity. We found that the Fe–O bond length, the d-band center gap of spin states, the magnetic moment of Fe site and *O_2_ as descriptors can accurately predict the ORR activity of Fe-N_4_ site. Furthermore, these descriptors and ORR activity of Fe-N_4_ site are mainly distributed in two regions with obvious difference, which greatly relate to the height of Fe 3*d* projected orbital in the Z direction. This work provides a new insight into the ORR activity of single-atom M-N-C catalysts.

## Abstract (web summary)

It is of high importance to understand the origin of single-atom Fe-N_4_ activity in oxygen reduction reaction. Here, the authors provide a model to understand the catalytic activity of Fe-N_4_ site from the spatial structure and energy level of the frontier orbitals by density functional theory calculations.

## Introduction

Single-atom Fe-N-C catalysts are considered a candidate to replace the platinum group metals in oxygen reduction reaction (ORR), due to its high activity, anti-toxicity, and metal atom utilization^1–4^. Advanced Fe-N-C materials show comparable ORR performance to that of benchmark Pt/C^3,5–7^. Both theoretical and experimental studies indicate that the Fe-N_4_ site is the active species of Fe-N-C catalysts^3,4,6,8–10^. However, the ORR activity of Fe-N_4_ site on different carbon supports varies greatly, and Fe-N_4_ ORR activity on curved carbon supports is usually orientation-dependence^11–15^. The essential of the difference and orientation-dependence of Fe-N_4_ ORR activity remains unclear. Therefore, understanding the ORR activity of the Fe-N_4_ site is crucial to further improve the ORR performance of Fe-N-C catalysts.

The intrinsic ORR activity of the Fe-N_4_ site relates to the electronic structure of carbon supports^7,16^. Controlling the electronic structure of carbon supports alters the interaction between the supports and the Fe-N_4_ site, resulting in the activity change of the Fe-N_4_ site^6,7,13,16–18^. However, the essential of the change in Fe-N_4_ ORR activity is still puzzling due to the complex factors affecting it. Some works reported that the electronic structure of carbon supports (electron-withdrawing/donating property) can affect the d-band center or d-orbital level of Fe site^17,19–21^. While, others claimed that it regulates the charge state of Fe site^6,22,23^. To understand the Fe-N_4_ ORR activity, many efforts have been made to explore the descriptors of Fe-N_4_ ORR activity^13,16,20,24–26^. For example, the full width at half-maximum of C 1*s* photoemission spectra^13^, d-band center^20^, and the electronegativity of the nearest neighbor atoms^24^, are considered to describe the ORR activity of the Fe-N_4_ site. However, these descriptors are difficult to understand the orientation-dependence and differences of ORR activity at the same Fe-N_4_ site on different carbon supports^11,12,14^. Therefore, elucidating the origin of ORR activity and exploring the universal descriptors of ORR activity on the Fe-N_4_ site are urgent.

In this work, we demonstrate that the hybridization among Fe 3*dz*^2^, 3*dyz* (3*dxz*), and O_2_ π* orbital is the origin of ORR activity on the Fe-N_4_ site by density functional theory (DFT) calculations. We established the relationship between the geometric, electronic structure, and ORR activity of the Fe-N_4_ site. From the geometric structure, the Fe–O bond length (L_Fe-O_) can accurately describe the ORR activity of the Fe-N_4_ site. From the electronic structure, the d-band center gap of spin states (Δd), the magnetic moment of the Fe site (M_Fe_), and *O_2_ (M_*O2_) are nearly linear in relationship to the ORR activity of the Fe-N_4_ site. We found that these descriptors and Fe-N_4_ ORR activity are located in two regions. We provided a model to elucidate the origin and correlation of these descriptors from the spatial structure and level of the frontier orbital. This model reveals that descriptors essentially reflect hybridization between Fe 3*dz*^2^, 3*dyz* (3*dxz*), and O_2_ π* orbital, which is related to the height of 3*d* orbital spatial distribution. This study offers new insights into the origin and descriptor of ORR activity on M-N-C catalysts and provides guidance for the design of high-performance ORR catalysts.

## Results

### Structural stability and ORR activity of Fe-N_4_ sites

**Fig. 1:**
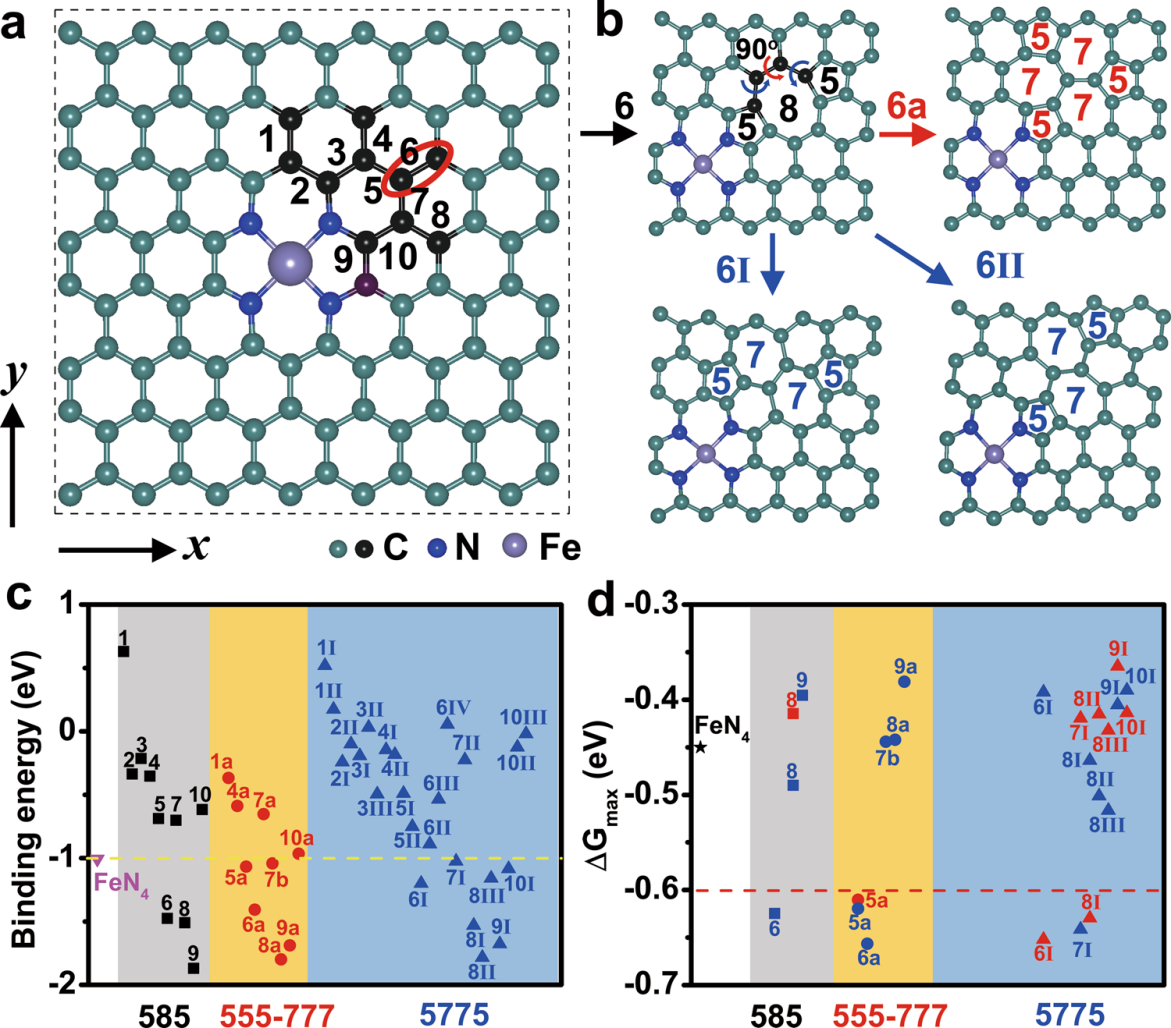
Modeling and performance evaluation of single-atom Fe-N_4_ sites. **a** Optimized structure of a perfect FeN_4_ site. **b** Divacancy defect models 6, 6a, 6I, and 6II. **c** The binding energy of Fe site. **d** The ORR activity of Fe-N_4_ sites. Blue and red represent upper and lower surface activity, respectively.

Here, we built the model of the Fe-N_4_ site embedded in the graphene slab (Fig. 1a). We introduced various divacancy defects around the Fe-N_4_ site to obtain the various sample of Fe-N_4_ ORR activity, since divacancy defects can modify the electronic structure of the carbon supports and also affect the ORR activity of the Fe-N_4_ site^13,16,22,27–29^. Then pentagon-octagon-pentagon (5-8-5) defects were constructed by selectively removing C–C bonds around the Fe-N_4_ site. Triple pentagon-triple heptagon (555-777) and pentagon-heptagon-heptagon-pentagon (5-7-7-5) defects were obtained by rotating the different C–C bonds (Fig. 1b). A total of 42 types of divacancy defects were constructed around the Fe-N_4_ site, including ten types of 5-8-5 (Supplementary Fig. 1), nine types of 555-777 (Supplementary Fig. 2), and 23 types of 5-7-7-5 (Supplementary Fig. 3) defects.

To evaluate the stability of single-atom Fe sites on carbon supports, we calculated the binding energy (E_bind_) of single-atom Fe sites (Fig. 1c). For the perfect Fe-N_4_ configuration, the binding energy of the single-atom Fe site is –1.01 eV, indicating that the configuration of the Fe-N_4_ site on carbon supports is stable. Under the control of divacancy defects, the binding energies of Fe sites range from –1.88 ~0.63 eV. Obviously, divacancy defects on the carbon supports can affect the stability of a single-atom Fe site. Among them, the relatively stable systems (E_bind_ <–1.01 eV) were selected as examples to study the ORR activity of Fe-N_4_ sites, which include 5-8-5 (6, 8, 9), 555-777 (5a, 6a, 7b, 8a, 9a), and 5-7-7-5 defect (6I, 7I, 8I, 8II, 8III, 9I, 10I). The ORR activity on the upper (blue) and lower (red) sides of Fe-N_4_ configurations were investigated due to surface asymmetry (Fig. 1d). We found the hydrogenation of *O_2_ is a potential-determining step (PDS) at all Fe-N_4_ configurations. Divacancy defect can regulate the free energy difference of this step on the Fe-N_4_ site (Supplementary Table 1). The maximal free energy difference (ΔG_max_) of ORR at the perfect FeN_4_ site is –0.45 eV, which is consistent with previous studies^3,6,30^. Among them, divacancy defects, such as 6, 5a, 6a, 6I, 7I, and 8I, can enhance the activity of the Fe-N_4_ site, with ∆G_max_ less than –0.60 eV (Fig. 1d). For the asymmetric surfaces, the ORR activity of the Fe-N_4_ site showed orientation-dependence and difference, such as 6I, 7I, 8I, etc. Besides, divacancy defects can control the electron-donating capability of the carbon support, ultimately affecting the charge state and the d-band center of the Fe site (Supplementary Fig. 4). However, these factors are difficult to elucidate the orientation-dependence of the ORR activity of the Fe-N_4_ site on an asymmetric surface.

### Factors of ORR activity at the Fe-N_4_ site

**Fig. 2:**
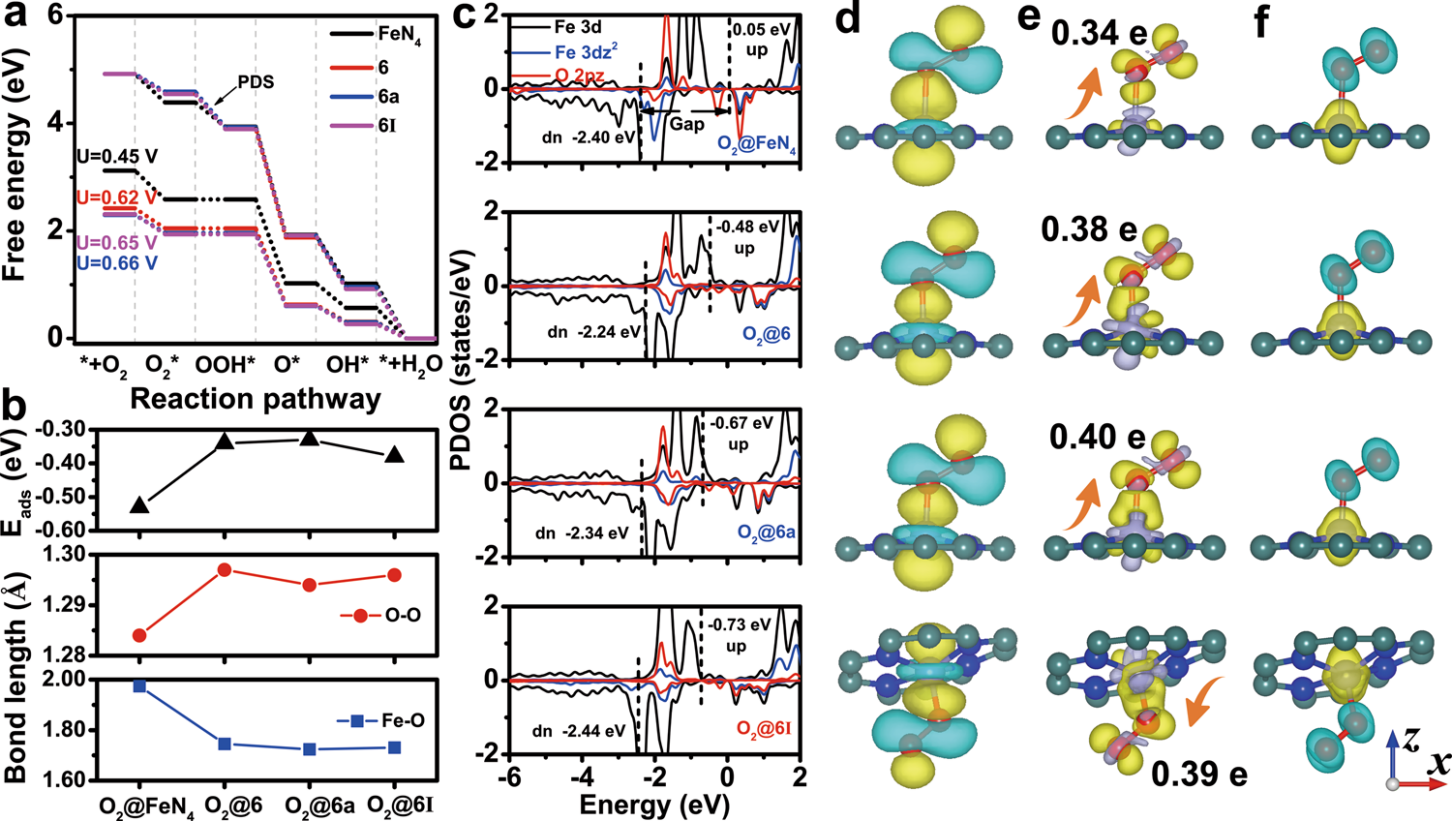
Theoretically study of the origin of Fe-N_4_ ORR activity. **a** Gibbs free energy diagrams of ORR on perfect FeN_4_ site, 6, 6a, and 6I. **b** Adsorption energy of *O_2_, O–O, and Fe–O bond length on the O_2_@FeN_4_, O_2_@6, O_2_@6a, and O_2_@6I. **c** Projected density of states of Fe 3*d*z^2^, O_2_ 2*p*z orbital, and d-band center of Fe 3*d* spin state. **d** Maximally localized Wannier functions of Fe 3*d*z^2^, O_2_ 2*p*z orbital. **e** Charge density difference and **f** spin density.

We investigated the ORR process of Fe-N_4_ configurations from energy, geometry, and electronic structure to explore the origin of Fe-N_4_ ORR activity. Here, we highlight the results of 6 (5-8-5), 6a (555-777), and 6I (5-7-7-5) models because they can improve the ORR activity of the Fe-N_4_ site with good thermodynamic stability (Supplementary Fig. 5). The free energy diagram of ORR shows that the ΔG value of *OOH formation on 6, 6a, and 6I are –0.62, –0.65, and –0.66 eV (Fig. 2a and Supplementary Fig. 6), respectively. Compared with the perfect FeN_4_ site (–0.45 eV), divacancy defects (6, 6a, and 6I) can facilitate *O_2_ conversion to *OOH intermediate. We found that the adsorption energy of *O_2_ with end-on1 and end-on2 configurations on the FeN_4_ site are –0.53 and –0.41 eV, respectively, and the adsorption energy of *O_2_ with side-on configuration on the FeN_4_ site is –0.01 eV (Supplementary Fig. 7). This result shows that the end-on adsorption is more favorable than the side-on adsorption at the FeN_4_ site. Among them, the adsorption energies of *O_2_ on 6, 6a, and 6I are –0.34, –0.33, and –0.38 eV (Fig. 2b), respectively, which are larger than that of the perfect FeN_4_ site (–0.53 eV). These results suggest the weak adsorption of *O_2_ on the Fe-N_4_ site is the key to enhance the ORR activity of the Fe-N_4_ site. Meanwhile, the O–O bond lengths on the O_2_@FeN_4_, O_2_@6, O_2_@6a, and O_2_@6I are 1.284, 1.297, 1.294, and 1.296 Å (Fig. 2b), respectively, which are longer than the bond length of O_2_ molecule (1.234 Å). Besides, the Fe–O bond lengths on the O_2_@FeN_4_, O_2_@6, O_2_@6a, and O_2_@6I are 1.974, 1.745, 1.724, and 1.731 Å (Fig. 2b), respectively. These results reveal that divacancy defects (6, 6a, and 6I) can enhance the interaction between *O_2_ and Fe site and promote the activation of *O_2_, thus increasing the Fe-N_4_ ORR activity.

We studied the electronic structure of the Fe-N_4_ site and O_2_ to understand the activation mechanism of *O_2_. Before the adsorption of O_2_, the projected density of state (PDOS) of the Fe site indicates that the Fe site has spin polarization with a spin magnetic moment of 2 μB. The occupied states of the Fe site near the Fermi level consist of localized 3*dxz*, 3*dx*^2^–*y*^2^, and 3*dz*^2^ (Supplementary Fig. 8). Compared to the PDOS of the FeN_4_ site, divacancy defects (6, 6a, and 6I) modified the intensity and distribution of Fe 3*d* states (especially 3*dxz* and 3*dz*^2^). For the O_2_ molecule, the PDOS of O_2_ also shows spin polarization with a spin magnetic moment of 2 μB. The anti-bonding orbitals (π*) of O_2_ are mainly composed of the O_2_ 2*p*y and 2*p*z orbitals (Supplementary Fig. 8). When O_2_ adsorb on the Fe-N_4_ site, the 2*p*z orbital in the O_2_ π* orbital split into the discrete level, and the intensity of localized Fe 3*d*z^2^ states is significantly reduced (Supplementary Fig. 9). A new hybrid state formed near the Fermi level, due to the hybridization between O_2_ 2*p*z and Fe 3*d*z^2^ orbital. In the O_2_@6, O_2_@6a, and O_2_@6I, the strong hybridization between Fe 3*d*z^2^, and O 2*p*z orbital lead to the significant discrete of the O_2_ 2*p*z orbital state (Fig. 2c), suggesting that the O_2_ molecules are better activated. Besides, there is also hybridization between the 2*p*y orbital of O_2_ and the 3*dyz* orbital of Fe (Supplementary Fig. 9). The d-band center of Fe spin-up and spin-down are close to each other due to hybridization (Fig. 2c and Supplementary Fig. 4). The d-band center gap of Fe spin state (Δd) of O_2_@FeN_4_, O_2_@6, O_2_@6a, and O_2_@6I are 2.45, 1.76, 1.67, and 1.70 eV (Fig. 2c), respectively, meaning the Δd is related to the activation of O_2_.

We also investigated the spatial distribution of Fe 3*d*z^2^ (3*d*yz) and O_2_ 2*p*z (2*p*y) orbital by the maximally localized Wannier functions (MLWF)^31^. The Wannier function of Fe 3*d*z^2^ and Fe 3*d*yz are located vertically and slant (Supplementary Figs. 10 and 11). O_2_ 2*p*z orbital Wannier function shows high mirror symmetry (Supplementary Fig. 12). When O_2_ is adsorbed on the Fe-N_4_ site, the Wannier function of Fe 3*d*z^2^ (3*d*yz) and O_2_ 2*p*z (2*p*y) shows the trend of orbital matching (Fig. 2d and Supplementary Figs. 13, 14). Wannier function of Fe 3*d*z^2^ orbital decreases with that of O_2_ 2*p*z orbital increases (Fig. 2d and Supplementary Fig. 13). Especially, the changes of Fe 3*d*z^2^ (3*d*yz) and O_2_ 2*p*z (2*py*) orbital Wannier functions on O_2_@6, O_2_@6a, and O_2_@6I are larger than that on O_2_@FeN_4_. These results demonstrate that divacancy defects (6, 6a, and 6I) can enhance the hybridization between Fe and O_2_ to facilitate the activation of O_2_.

Generally, the filling of electrons in the O_2_ π* orbital is the beginning of the activation of O_2_. Charge density difference and Bader charge analysis reveal *O_2_ gain 0.34, 0.38, 0.40, and 0.39 *e* from perfect FeN_4_, 6, 6a, and 6I (Fig. 2e), respectively. The electron-gaining region (yellow region) of the *O_2_ mainly distributes in the π* orbitals of O_2_, which is consistent with the distribution region of the Wannier function (Fig. 2d and Supplementary Figs. 13, 14), reflecting that the electron transfer between Fe 3*d*z^2^ (3*d*yz) and O_2_ π* orbitals. Furthermore, due to electrons transfer between Fe sites and O_2_, the Fe 3*d*z^2^ (3*d*yz) and O_2_ π* orbitals are broken, thus changing the magnetic moment of the Fe site and O_2_. Spin density analysis show divacancy defects (6, 6a, and 6I) hardly change the spin density of Fe sites before O_2_ adsorption (Supplementary Fig. 15). After O_2_ adsorption, the spin density is mainly localized at the Fe site and *O_2_ has the opposite direction (Fig. 2f). The spin density of the Fe site and *O_2_ in the O_2_@6, O_2_@6a, and O_2_@6I is weaker than that in O_2_@FeN_4_ (Fig. 2f). Therefore, the origin of O_2_ activation is the hybridization of the Fe 3*d*z^2^ (3*d*yz) and *O_2_ 2*p*z (2*p*y) orbitals, which results in electron transfer from the Fe site to *O_2_, breaking the Fe 3*d*z^2^ (3*d*yz) and O_2_ 2*p*z (2*p*y) orbitals and reducing Δd and magnetic moments.

### Descriptors of ORR activity at Fe-N_4_ sites

**Fig. 3:**
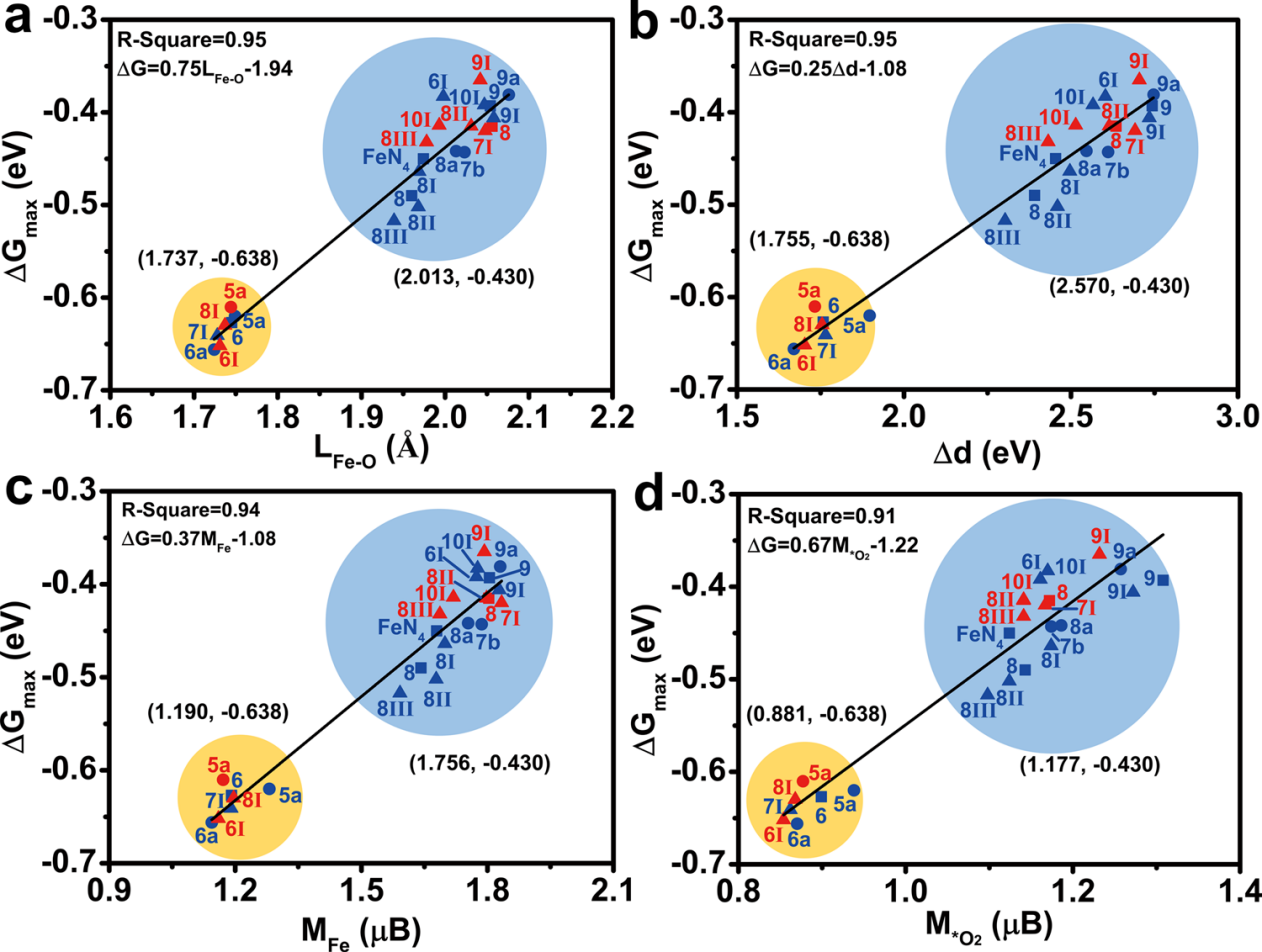
Exploring the descriptors of Fe-N_4_ ORR activity. Correlation between the **a** L_Fe–O_ (Fe–O bond length), **b** Δd (the d-band center gap of Fe spin state), **c** M_Fe_ (the spin magnetic moment of Fe), **d** M_*O2_ (the spin magnetic moment of *O_2_) and the ORR activity of Fe-N_4_ site. Blue and red represent upper and lower surface activity, respectively. Golden and blue circles represent the active and inactive Fe-N_4_ site, respectively.

As mentioned above, the adsorption energy of *O_2_, the bond length, the charge state, Δd, and the magnetic moment may be related to the ORR activity of the Fe-N_4_ site (Supplementary Table 2). To gain accurate descriptors for the ORR activity of the Fe-N_4_ site, we investigated the correlation between these factors and ORR activity (Fig. 3 and Supplementary Figs. 16–18). According to the free energy diagram, the adsorption energy of *O_2_ may be related to ORR activity. The adsorption energy of *O_2_ at the Fe-N_4_ site ranges from –0.6 to –0.1 eV under the regulation of divacancy defects (Supplementary Fig. 16). The linear fitting results reveal that the coefficient of determination R-square between the adsorption energy of *O_2_ and the ORR activity of the Fe-N_4_ site is 0.15. No direct correlation between the adsorption energy of *O_2_ and ORR activity at the Fe-N_4_ site. From the geometric structure, the O–O bond length (L_O-O_) is related to the activation of O_2_ molecules, and the Fe–O bond length (L_Fe-O_) can reveal the strength of the interaction between *O_2_ and Fe-N_4_ site. Herein, we found that the O–O bond length is distributed between 1.264–1.298 Å by regulating divacancy defects (Supplementary Fig. 17). When the O–O bond length is larger than 1.290 Å, and the free energy difference for hydrogenation of *O_2_ is less than –0.6 eV. The coefficient of determination R-square between the O–O bond length and Fe-N_4_ ORR activity is 0.81, with the relevant formula being ΔG = –9.93L_O–O_ + 12.3 (Supplementary Fig. 17). Besides, the length of the Fe–O bond is distributed in stages (Fig. 3a). For the active Fe-N_4_ site (golden circle), the average Fe–O bond length is around 1.737 Å. For the inactive Fe-N_4_ site (blue circle), the average Fe–O bond length is near 2.013 Å. The coefficient of determination R-square between the Fe–O bond length and Fe-N_4_ ORR activity is 0.95 (Fig. 3a), indicating the Fe–O bond length can accurately describe the ORR activity of the Fe-N_4_ site with the relevant formula ΔG = 0.75L_Fe–O_ + 1.94.

From the electronic structure, the charge state of the Fe site (*O_2_) can reflect the electrons transfer between Fe and O_2_, which may have a potential relationship with Fe-N_4_ ORR activity (Supplementary Fig. 18). The Bader charge state of Fe and *O_2_ are –1.22 to –1.13 *e* and 0.24 to 0.41 *e* (Supplementary Fig. 18), respectively, due to the regulation of divacancy defect. The linear fitting results show that the coefficient of determination R-square between the charge state of the Fe site (*O_2_) and the ORR activity of the Fe-N_4_ site is 0.10 (0.58), indicating that they are difficult to describe the ORR activity of Fe-N_4_ site (Supplementary Fig. 18). In addition, Δd (Fig. 3b), M_Fe_ (Fig. 3c), and M_*O2_ (Fig. 3d) are also characteristic symbols for the change of Fe and *O_2_ electronic structure. We found that Δd, M_Fe_, and M_*O2_ are also distributed in stages (Fig. 3b–d). The smaller their values, the higher the ORR activity of the Fe-N_4_ site. After the adsorption of O_2_, the average values of Δd, M_Fe_, and M_*O2_ on the active Fe-N_4_ site (golden circle) are 1.755 eV, 1.190 μB, and 0.881 μB, respectively. Correspondingly, the average of ΔG is –0.638 eV. For the inactive Fe-N_4_ sites (blue circle), the average of ΔG, Δd, M_Fe_, and M_*O2_ are –0.430 eV, 2.570 eV, 1.756 μB, and 1.177 μB, respectively. These fitting results show that the correlation between Δd, M_Fe_, M_*O2_, and ΔG is nearly linear. The R-squares are 0.95, 0.94, and 0.91 (Fig. 3b–d), respectively, indicating Δd, M_Fe_, M_*O2_ are promising ORR activity descriptors for the Fe-N_4_ site. The relevant formulas are ΔG = 0.25Δd – 1.08, ΔG = 0.37M_Fe_ – 1.08, ΔG = 0.67 M_*O2_ – 1.22, respectively.

To generalize the correlations between electronic structure and Fe-N_4_ ORR activity, we calculated the electronic structure of O_2_@Fe-N_4_ by using other theoretical approximations, including local density approximation (LDA (CA))^32,33^, Perdew–Wang 91 (GGA (PW91))^34^, strongly constrained and appropriately normed semilocal density functional (meta-GGA (SCAN))^35^, and Heyd–Scuseria–Ernzerhof screened hybrid density functional (HSE06)^36,37^. The results show that the type of DFT exchange-correlation functional can affect the absolute value of the spin magnetic moment of Fe (M_Fe_), the spin magnetic moment of *O_2_ (M_*O2_), and the d-band center gap of spin state (∆d), but the variation tendency of the value is consistent (Supplementary Figs. 19–22 and Supplementary Tables 3–5). There is still a nearly linear correlation between electronic structure and Fe-N_4_ ORR activity in the theoretical framework of LDA (CA) + U, GGA (PW91) + U, meta-GGA (SCAN), and HSE06 (Supplementary Figs. 20–22). M_Fe_, M_*O2_, and Δd are still distributed in stages. For the LDA (CA) + U, GGA (PW91) + U, meta-GGA (SCAN), and HSE06, the R-Square between M_Fe_ and ΔG are 0.93, 0.95, 0.91, and 0.91 (Supplementary Fig. 20), respectively. In addition, M_*O2_ and Δd were also highly correlated with ΔG (Supplementary Figs. 21 and 22). Therefore, the correlation between the electronic structure and the Fe-N_4_ ORR activity remains valid in other theoretical approximations.

### Correlations between the descriptors of ORR activity at Fe-N_4_ sites

**Fig. 4:**
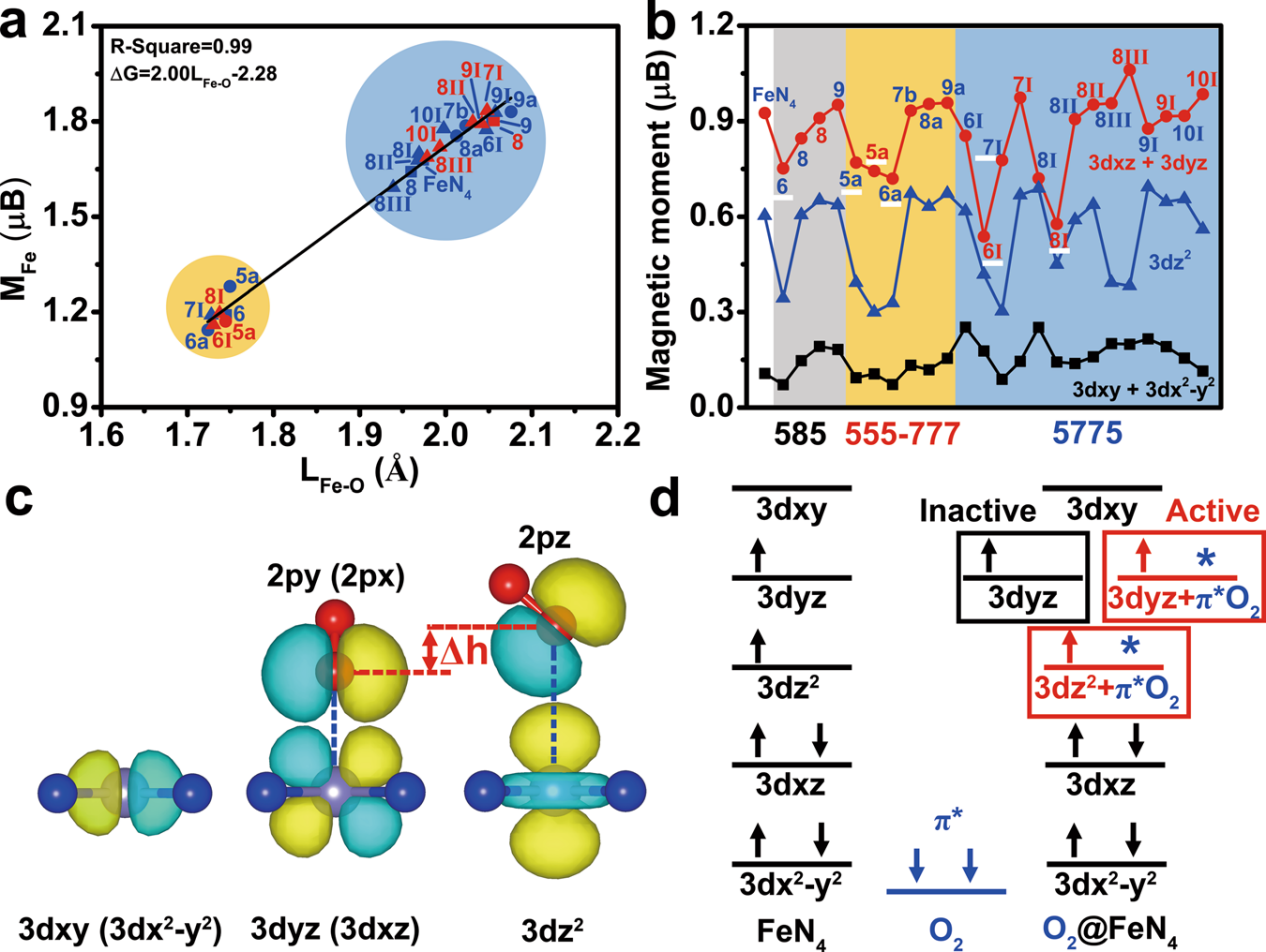
Correlation mechanisms between descriptors. **a** Correlation between the Fe–O bond length and magnetic moment of Fe site. **b** The magnetic moment of the 3*dxy* + 3*dx*^2^ – *y*^2^, 3*dyz* + 3*dxz* and 3*dz*^2^ orbital at the Fe site. **c** The spatial distribution of Fe 3*dxy* (3*dx*^2^ – *y*^2^), 3*dyz* (3*dxz*), 3*dz*^2^, and O_2_ 2π* orbital. **d** The schematic frontier orbital diagram of Fe-N_4_, O_2_, and O_2_@Fe-N_4_.

We explored the correlation between these descriptors. Obviously, there is a nearly linear relationship between Δd, M_Fe_, M_*O2_, and the Fe–O bond length, with the R-squares of 0.98, 0.99, and 0.94 (Fig. 4a and Supplementary Fig. 23), respectively, indicating that these descriptors can reflect the origin of Fe-N_4_ ORR activity. To reveal the essence of descriptors, we analyzed the projected magnetic moment of Fe, the spatial structure of Fe 3*d* and O_2_ π* orbital, and the schematic frontier orbital level diagram.

The projected magnetic moment of Fe fluctuates obviously, due to the regulation of the divacancy defects (Fig. 4b). Only when the magnetic moments of 3*dz*^2^ and 3*dyz* + 3*dxz* decrease at the same time, the Fe-N_4_ site shows higher ORR activity. This result suggests that the ORR activity at the active Fe-N_4_ site is attributed to the strong hybridization of Fe 3*dz*^2^–*O_2_ 2*pz* and Fe 3*dyz*–*O_2_ 2*py* (Fe 3*dxz*–*O_2_ 2*px*).

The spatial distribution of Fe 3*d* and O_2_ π* orbital can elucidate the correlation between the Fe–O bond length and M_Fe_. As shown in Fig. 4c, the 3*dxy* (3d*x*^2^–*y*^2^), 3*dyz* (3*dxz*) and 3*dz*^2^ are different heights in the Z direction. When the Fe–O bond is long, the activation of O_2_ attribute to the hybridization between Fe 3*dz*^2^ and O_2_ 2*pz* orbital, which leads to the decrease of the projected magnetic moments of Fe 3*dz*^2^. When the Fe–O bond is short, the activation of O_2_ is additionally improved by the hybridization of Fe 3*dyz* (3*dxz*) and 2*py* (2*px*) orbitals. Meanwhile, the projected magnetic moments of Fe 3*dz*^2^ and 3*dyz* (3*dxz*) are significantly reduced. The Fe 3*dxy* and 3*dx*^2^–*y*^2^ have weak hybridization with O_2_ π* orbital due to their low spatial distribution in the Z direction. Thereby the projected magnetic moments of 3*dxy* and 3*dx*^2^–*y*^2^ vary only slightly.

The schematic frontier orbital diagram of O_2_@Fe-N_4_ can reveal the differences in O_2_ activation (Fig. 4d)^38^. Before the adsorption of O_2_, the Fe 3*d* orbital splits into an empty 3*dxy*, singly occupied 3*dyz*, 3*dz*^2^ type orbitals, and occupied 3*dxz*, 3*dx*^2^–*y*^2^ orbitals, due to Fe 3*d* orbital hybridization with the carbon support. For the O_2_@Fe-N_4_, the differences in O_2_ activation is attributed to the degree of hybridization of Fe 3*dz*^2^ and 3*dyz* (or 3*dxz*) with O_2_ π* orbital. For the active Fe-N_4_ site, the strong hybridization of 3*dz*^2^ and 3*dyz* (or 3*dxz*) with O_2_ π* orbital promote the O_2_ activation. For the inactive Fe-N_4_ site, the strong hybridization of 3*dz*^2^ with O_2_ π* orbital and negligible hybridization of 3*dyz* (or 3*dxz*) with O_2_ π* orbital lead to the poor O_2_ activation. The change of Δd, M_Fe_, and M_*O2_ are also due to the above factors. Therefore, the Fe-N_4_ ORR activity is origin from the activation of O_2_, that is, the hybridization between Fe 3*dz*^2^ (3*dyz* or 3*dxz*) and *O_2_ π* orbital. The Fe–O bond length, Δd, M_Fe_, and M_*O2_ are the specific symbols describing hybridization.

## Discussion

In this work, we investigated the origin and descriptors of ORR activity on the Fe-N_4_ site using DFT calculation. We explored divacancy defects on carbon supports that could modulate the ORR activity of the Fe-N_4_ site. The first electron step (*O_2_ + H^+^ + e → *OOH) is the potential-determining step (PDS) on all Fe-N_4_ configurations. Fe-N_4_ site on 6, 5a, 6a, 6I, 7I, and 8I showed high ORR activity with ΔG_max_ less than –0.60 eV. The ORR activity of Fe-N_4_ sites on the asymmetric surface showed orientation-dependence. We demonstrated that the hybridization between Fe 3*dz*^2^, 3*dyz* (3*dxz*), and O_2_ π* orbitals is the origin of Fe-N_4_ ORR activity by studying the electronic structure. The Fe–O bond length, Δd, M_Fe_, and M_*O2_ show staged differences, which can be used as descriptors to accurately describe the ORR activity of the Fe-N_4_ site. There is a nearly linear correlation between Δd, M_Fe_, M_*O2_, and the Fe–O bond length from geometry and electron structure. Importantly, we elucidate that the Fe–O bond length, Δd, M_Fe_, and M_*O2_ are essentially hybrids between Fe 3*dz*^2^ (3*dyz*) and O_2_ π* orbitals from the spatial distribution and energy level of the frontier orbital. The height of the Fe 3*d* projected orbital is related to the activation of O_2_. For the active Fe-N_4_ sites, the strong hybridization of 3*dz*^2^ and 3*dyz* (or 3*dxz*) with O_2_ π* orbital results in the short Fe–O bond length (1.737 Å), low Δd (1.755 eV), M_Fe_ (1.190 μB), and M_*O2_ (0.881 μB). For the inactive Fe-N_4_ site, the strong hybridization of 3*dz*^2^ with O_2_ π* orbital and the negligible hybridization of 3*dyz* (3*dxz*) with O_2_ π* orbital lead to the long Fe–O bond length (2.013 Å), high Δd (2.570 eV), M_Fe_ (1.756 μB), and M_*O2_ (1.177 μB). This work offers insights into the Fe-N_4_ ORR activity and provides guidelines for designing the high-performance Fe-N-C catalysts.

## Methods

### DFT calculations

Our spin-polarized density functional theory simulation was calculated by using the Vienna ab initio simulation package (VASP)^39^. The PAW potentials and the generalized gradient approximation (GGA) of Perdew–Burke–Ernzerhof (PBE) were employed to describe the interaction of electron-ion and the electron-electron exchange and correlation functional, respectively^40,41^. After the test, the cutoff energy was set at 400 eV, and Monkhorst–Pack mesh with a 2 × 2 × 1 grid was used in our calculations after testing (Supplementary Fig. 24). van der Waals (VDW) forces were corrected with the D2 method of Grimme^42^. The convergence criterion was set at 0.02 eV Å^−1^ for the force and 1 × 10^−5^ eV per atom for energy. We used the correlation energy (U) of 4 eV and the exchange energy (J) of 1 eV for Fe 3*d* orbitals^43,44^. We built a super-cell (7 × 8) carbon substrate as a model, which includes 106 C atoms, 4 N atoms, and 1 Fe atom. The lattice parameters of this slab are a = 17.31 Å and b = 16.98 Å after optimization. The lattice constants are optimized again due to the introduction of divacancy defects in the system. The vacuum layer was set at 15 Å. Maximally localized Wannier functions (MLWFs) methods are applied by Wannier90 to calculate the spatial distribution of Fe 3*d* and O_2_ 2*p* orbital^31,45–47^. We also considered the effect of solvent on the ORR activity of the Fe-N_4_ site by employing an implicit solvent model^48,49^. To confirm the generalization validity of the correlation between electronic structure and Fe-N_4_ ORR activity, local density approximation with the Ceperly–Alder functional (LDA (CA))^32,33^, Perdew–Wang 91 (GGA (PW91))^34^, strongly constrained and appropriately normed semilocal density functional (meta-GGA (SCAN))^35^, and Heyd–Scuseria–Ernzerhof screened hybrid density functional (HSE06) were used to evaluate the electronic structure^36,37^. In the framework of LDA (CA) and GGA (PW91), we also consider the Coulomb interaction between Fe 3*d* orbital electrons, and the Hubbard U value is 3 eV. First-principles Born–Oppenheimer molecular dynamics (BOMD) simulation was performed in the canonical ensemble with Nosé–Hoover heat bath schemes^50,51^. During the simulation, spin polarization is not considered, and the Brillouin-zone integrations were performed using the Gamma-point grid. The total simulation time is 5 ps with a time step of 1 fs at 300 K. We also used the VESTA package to visualize the structures and charge density differences^52^.

The binding energy of the Fe site (E_*bind*_) on the carbon supports can be calculated by

1 $${{{{{{\rm{E}}}}}}}_{bind}={{{{{{\rm{E}}}}}}}_{{{{{{\rm{Fe}}}}}}@{{{{{\rm{sub}}}}}}}-{{{{{{\rm{E}}}}}}}_{{{{{{\rm{sub}}}}}}}-{{{{{{\rm{E}}}}}}}_{{{{{{\rm{Fe}}}}}}-{{{{{\rm{bulk}}}}}}}$$

where E_Fe@sub_ is the total energy of the Fe atom embedded in the carbon supports; E_sub_ and E_Fe_ are the energies of the substrate and single Fe atom in the Fe bulk, respectively.

The Gibbs free energy can be expressed as

2 $$\Delta {{{{{\rm{G}}}}}}=\Delta {{{{{\rm{E}}}}}}+\Delta {{{{{\rm{ZPE}}}}}}-{{{{{\rm{T}}}}}}\cdot\Delta {{{{{\rm{S}}}}}}$$

where ∆E, ∆ZPE, and ∆S are the reaction energy calculated by the DFT methods, the changes in zero-point energies, and the entropy during the reaction, respectively^53–55^. T is the temperature (298.15 K, in our work).

The d-band center gap of spin state (∆d) can be defined as

3 $$\Delta {{{{{\rm{d}}}}}}=|{\varepsilon }_{{{{{{\rm{up}}}}}}}-{\varepsilon }_{{{{{{\rm{dn}}}}}}}|=\left|\frac{{\int }_{-\infty }^{+\infty }{n}_{{{{{{\rm{up}}}}}}}(\varepsilon )\varepsilon d\varepsilon }{{\int }_{-\infty }^{+\infty }{n}_{{{{{{\rm{up}}}}}}}(\varepsilon )d\varepsilon }-\frac{{\int }_{-\infty }^{+\infty }{n}_{{{{{{\rm{dn}}}}}}}(\varepsilon )\varepsilon d\varepsilon }{{\int }_{-\infty }^{+\infty }{n}_{{{{{{\rm{dn}}}}}}}(\varepsilon )d\varepsilon }\right|$$

where ε_up_ is the d-band center of the Fe 3*d* spin-up projected density of states. And ε_dn_ is the d-band center of the Fe 3*d* spin-down projected density of states^56^.

The magnetic moment (M) is defined as

4 $${{{{{\rm{M}}}}}}={{{{{\rm{N}}}}}}({{{{{\rm{spin}}}}}}\mbox{-}{{{{{\rm{up}}}}}})-{{{{{\rm{N}}}}}}({{{{{\rm{spin}}}}}}\mbox{-}{{{{{\rm{down}}}}}})$$

5 $${{{{{\rm{M}}}}}}={{{{{\rm{M}}}}}}({{{{{\rm{3\it dxy}}}}}})+{{{{{\rm{M}}}}}}({{{{{{\rm{3\it dx}}}}}}}^{2}-{{{{{\it{y}}}}}}^{2})+{{{{{\rm{M}}}}}}({{{{{\rm{3\it dyz}}}}}})+{{{{{\rm{M}}}}}}({{{{{\rm{3\it dxz}}}}}})+{{{{{\rm{M}}}}}}({{{{{{\rm{3\it dz}}}}}}}^{2})$$

where N(spin-up) and N(spin-down) represent the number of electrons in the spin-up and spin-down occupied state, respectively^57,58^. M(3*dxy*), M(3*dx*^2^ – *y*^2^), M(3*dyz*), M(3*dxz*), and M(3*dz*^2^) represent the projected of magnetic moments on Fe 3*dxy*, 3*dx*^2^–*y*^2^, 3*dyz*, 3*dxz*, and 3*dz*^2^, respectively.

## Supplementary information

Supplementary Information

## Data Availability

The data that support the findings of this study are available from the corresponding author on reasonable request.

## Code availability

The computational codes used in this work are available from the corresponding author on reasonable request.
