# Supplementary material for: Insights into the activity of single-atom Fe-N-C catalysts for oxygen reduction reaction
Source: Nat Commun. 2022 Apr 19;13:2075. doi: 10.1038/s41467-022-29797-1 (PMC9018836; doi:10.1038/s41467-022-29797-1)
Supplement: Supplementary file 1 — Supplementary Information [file 41467_2022_29797_MOESM1_ESM.pdf]

Supplementary Information for

Insights into the activity of single-atom Fe-N-C catalysts for oxygen reduction reaction

Kang Liu[1,2], Junwei Fu[1], Yiyang Lin[1], Tao Luo[1], Ganghai Ni[1], Hongmei Li[1], Zhang Lin[2], and Min Liu[1*]

[1] Hunan Joint International Research Center for Carbon Dioxide Resource Utilization, School of Physics and Electronics, Central South University, Changsha 410083, Hunan, P. R. China

[2] School of Metallurgy and Environment, Central South University, Changsha 410083, Hunan, P. R. China

Correspondence and requests for materials should be addressed to M. Liu. (E-mail: minliu@csu.edu.cn)

Results and Discussion

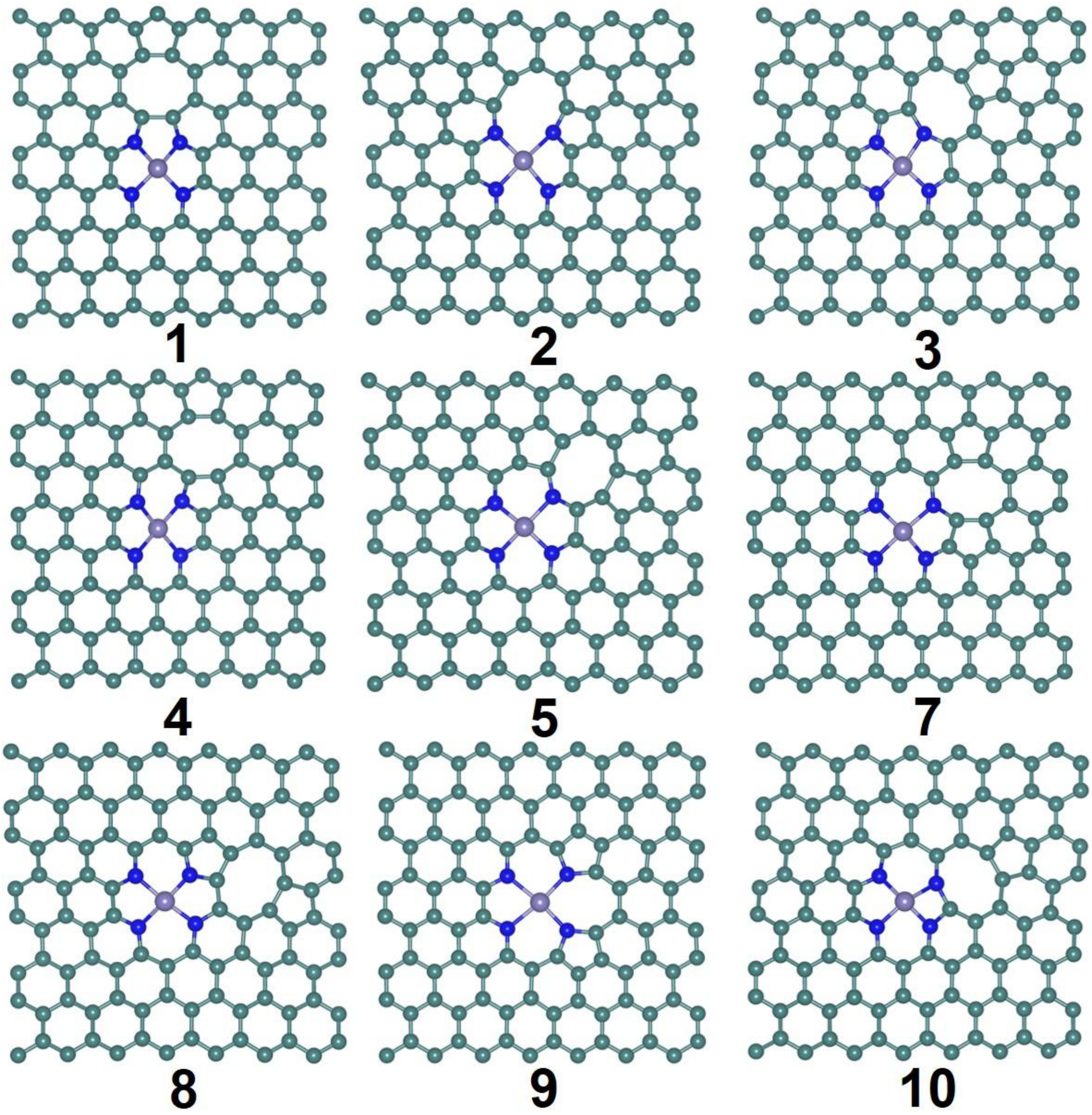

Supplementary Fig. 1 Optimized structure of 5-8-5 divacancy defects.

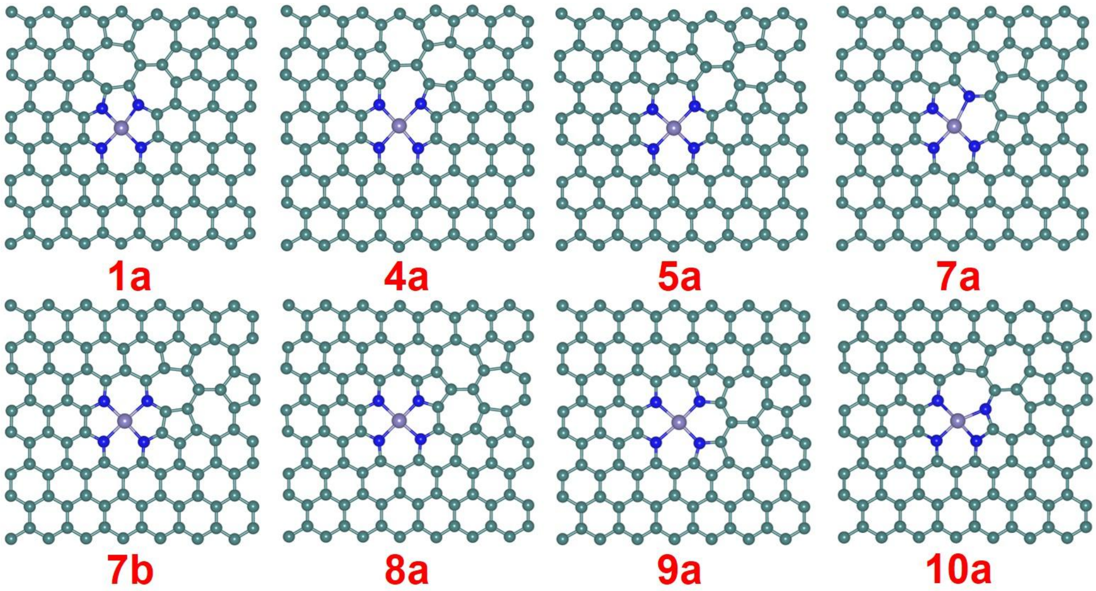

Supplementary Fig. 2 Optimized structure of 555-777 divacancy defects.

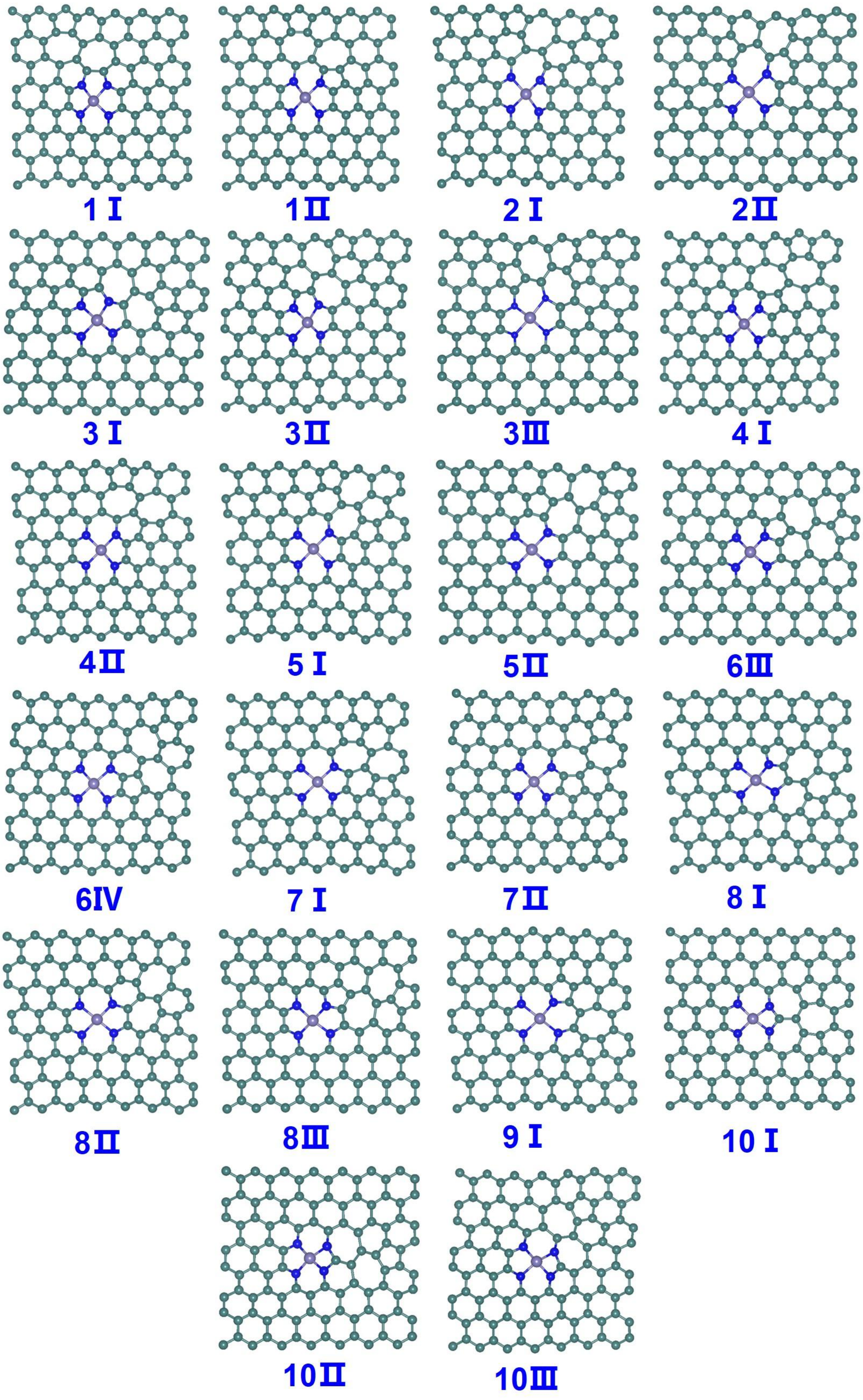

Supplementary Fig. 3 Optimized structure of 5-7-7-5 divacancy defects.

Supplementary Table 1. The free energy data of ORR on Fe-N_4 sites.

	O_2	*O_2	*OOH	O*	*OH	H_2O
FeN_4	4.920 (eV)	**4.386** (eV)	**3.936** (eV)	1.925 (eV)	1.016 (eV)	0 (eV)
6	4.920	**4.548**	**3.921**	1.879	0.934	0
8	4.920	**4.577**	**4.087**	2.084	1.156	0
8	4.920	**4.627**	**4.212**	2.251	1.250	0
9	4.920	**4.593**	**4.200**	2.344	1.226	0
5a	4.920	**4.595**	**3.975**	2.010	1.032	0
5a	4.920	**4.813**	**4.203**	2.120	1.099	0
6a	4.920	**4.588**	**3.932**	1.916	0.960	0
7b	4.920	**4.422**	**3.979**	2.036	1.059	0
8a	4.920	**4.421**	**3.979**	1.990	1.004	0
9a	4.920	**4.477**	**4.096**	2.178	1.117	0
6I	4.920	**4.428**	**4.036**	2.055	1.026	0
6I	4.920	**4.544**	**3.892**	1.916	0.919	0
7I	4.920	**4.511**	**3.870**	1.915	0.885	0
7I	4.920	**4.379**	**3.959**	2.038	0.984	0
8I	4.920	**4.399**	**3.935**	1.934	0.931	0
8I	4.920	**4.651**	**4.021**	2.021	0.986	0
8II	4.920	**4.388**	**3.886**	1.911	0.890	0
8II	4.920	**4.396**	**3.981**	2.036	0.994	0
8III	4.920	**4.369**	**3.852**	1.829	0.836	0
8III	4.920	**4.339**	**3.907**	1.909	0.937	0
9I	4.920	**4.559**	**4.153**	2.236	1.171	0
9I	4.920	**4.520**	**4.155**	2.206	1.147	0
10I	4.920	**4.422**	**4.039**	2.159	1.268	0
10I	4.920	**4.463**	**4.049**	2.008	1.012	0

Notes: The blue (red) data is the ORR activity of the upper (lower) surface of the model. The bold data are associated with the value of the potential-determining step (PDS).

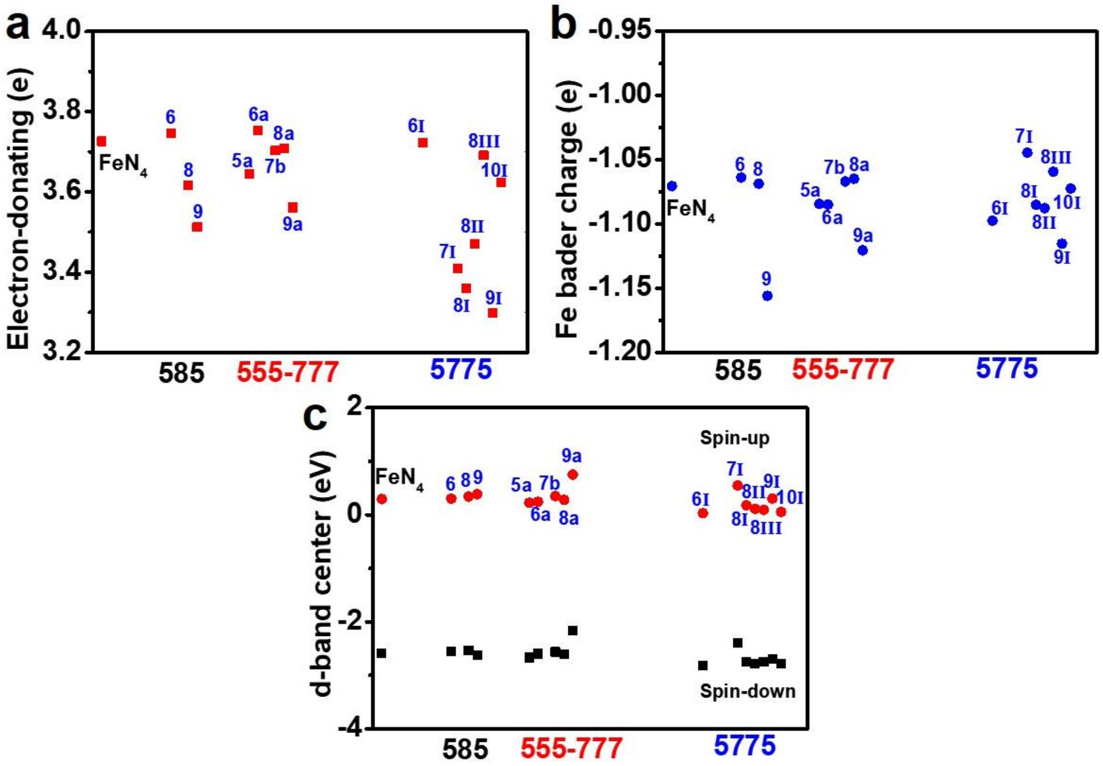

Supplementary Fig. 4 The electronic structure of single-atom Fe site. a The electron-donating capability of the carbon support. **b** The Bader charge state of Fe site. **c** The d-band center of Fe site.

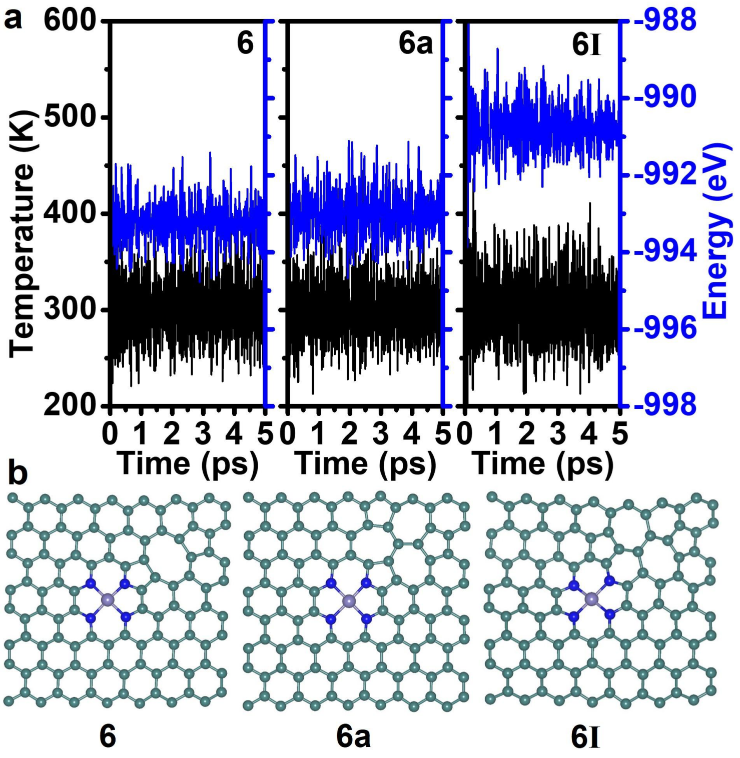

Supplementary Fig. 5 Thermodynamic stability of Fe-N$_4$ model with divacancy. a The relationship between temperature, energy and time for AIMD simulations of 6, 6a and 6I. **b** After run 5 ps with a time step of 1 fs, the top views of the structure of 6, 6a and 6I under 300 K.

The thermodynamics stability of Fe-N$_4$ sites on the 6, 6a and 6I was verified by Born-Oppenheimer molecular dynamics (BOMD) simulation. The oscillation of energy and temperature indicates Fe-N$_4$ sites on the 6, 6a and 6I are in equilibrium states (Supplementary Fig. 5a). And their geometries are well maintained after 5 ps with a time step of 1 fs at 300 K using Nosé–Hoover heat bath schemes (Supplementary Fig. 5b). Thus, Fe-N$_4$ sites on 6, 6a and 6I have excellent activity and good stability, which maybe exist stably in Fe-N-C catalysts.

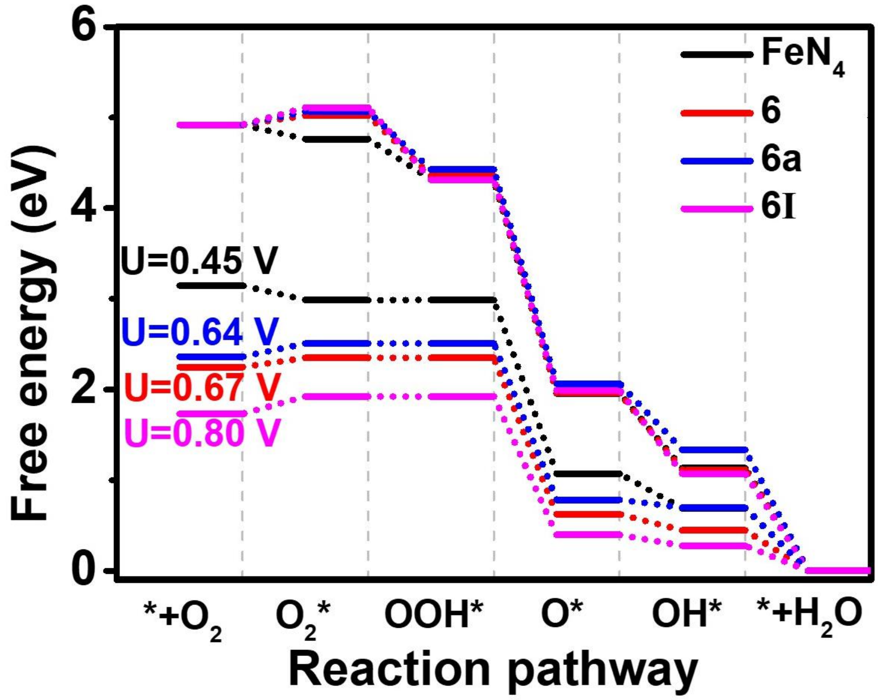

Supplementary Fig. 6 Gibbs free energy diagrams of ORR on perfect FeN_4 site, 6, 6a and 6I by employing implicit solvent model.

We also considered the effect of solvent on the ORR activity of Fe-N_4 site by employing implicit solvent model. The results also demonstrate that Fe-N_4 sites on the 6, 6a and 6I have excellent activation of $*O_2$, and the ΔG_{max} are –0.67, –0.64 and –0.80 eV (Supplementary Fig. 6), respectively.

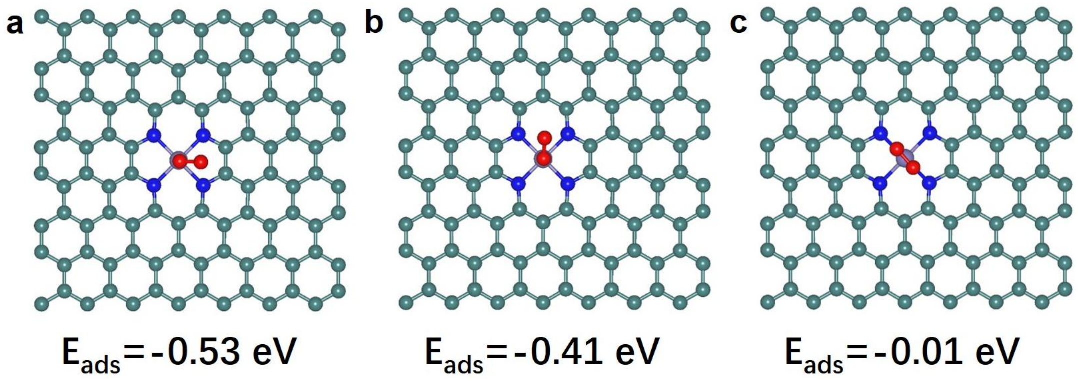

Supplementary Fig. 7 Adsorption configuration. The adsorption energy of $^*O_2$ with **a** end-on1, **b** end-on2, and **c** side-on configuration at the FeN_4 site.

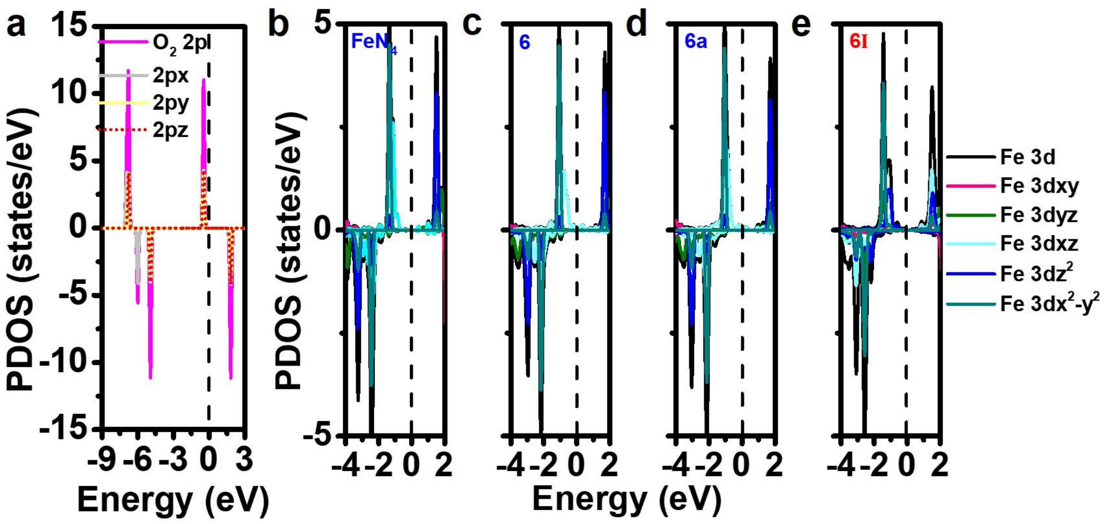

Supplementary Fig. 8 Projected density of states (PDOS). PDOS of **a** O_2, and PDOS of Fe site at **b** perfect FeN_4, **c** 6, **d** 6a, and **e** 6I.

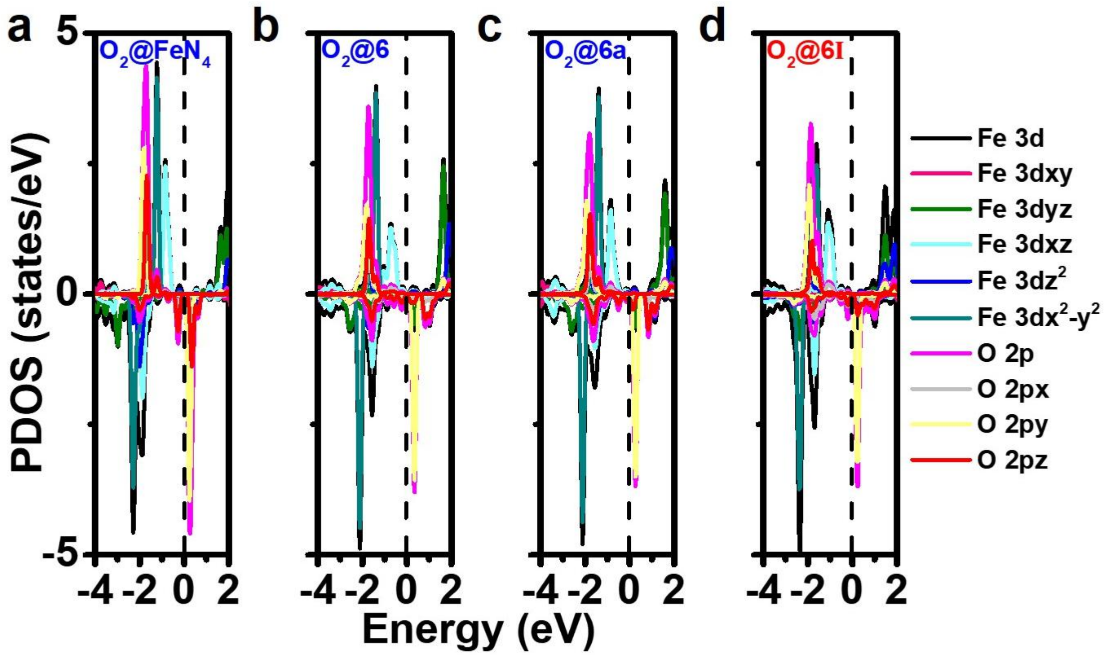

Supplementary Fig. 9 Projected density of states. PDOS of Fe site and O_2 after adsorption of O_2 at **a** O_2@FeN_4, **b** O_2@6, **c** O_2@6a and **d** O_2@6I.

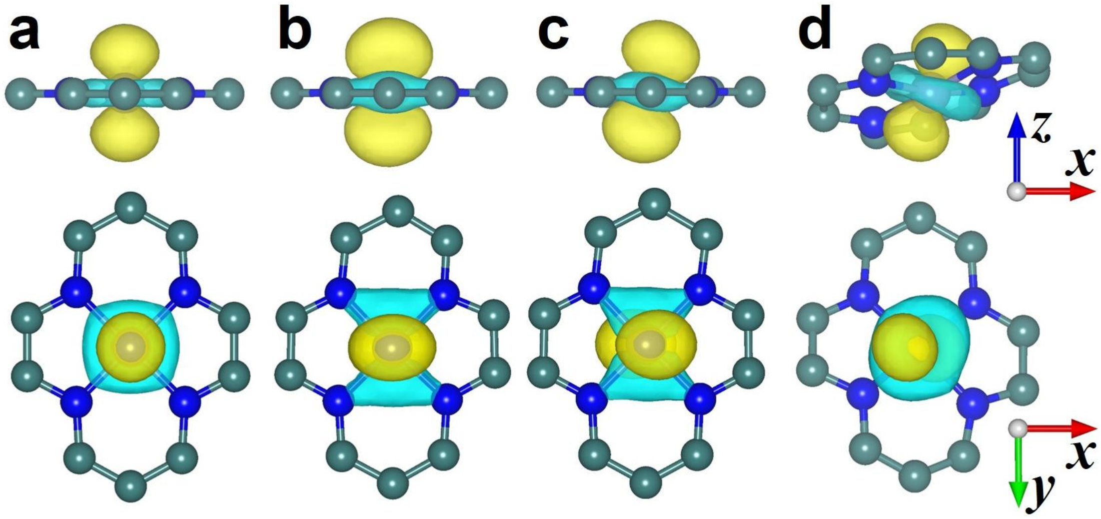

Supplementary Fig. 10 Wannier function. Top and side view of Fe 3dz^2 Wannier function before O_2 adsorbed on **a** perfect FeN_4, **b** 6, **c** 6a, and **d** 6I.

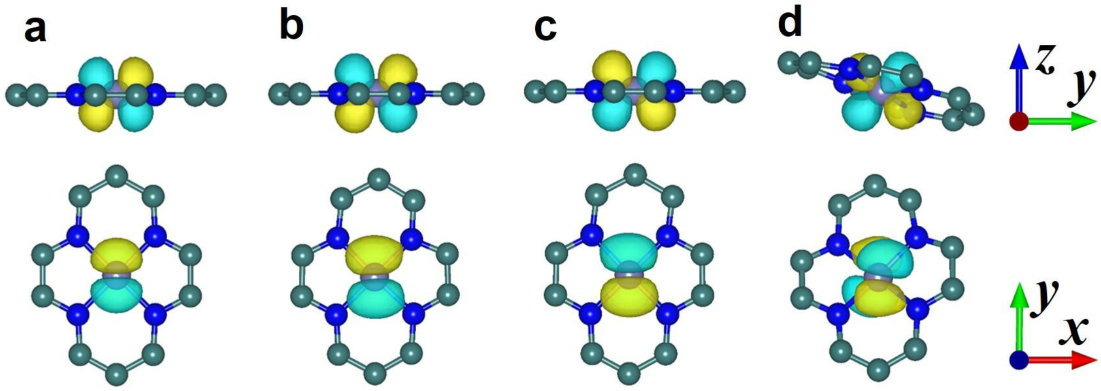

Supplementary Fig. 11 Wannier function. Top and side view of Fe 3dyz Wannier function at **a** perfect FeN_4, **b** 6, **c** 6a, and **d** 6I.

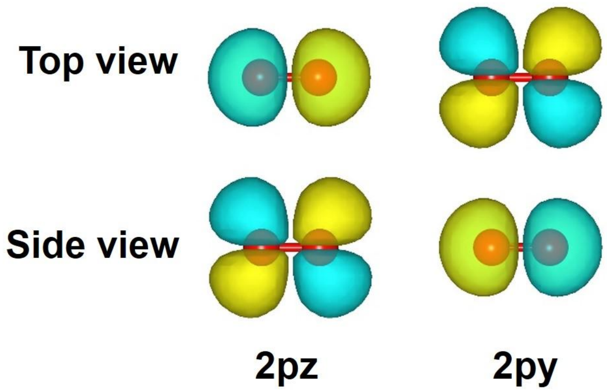

Supplementary Fig. 12 Calculated Wannier function of the 2pz and 2py orbital in the free O_2 molecule.

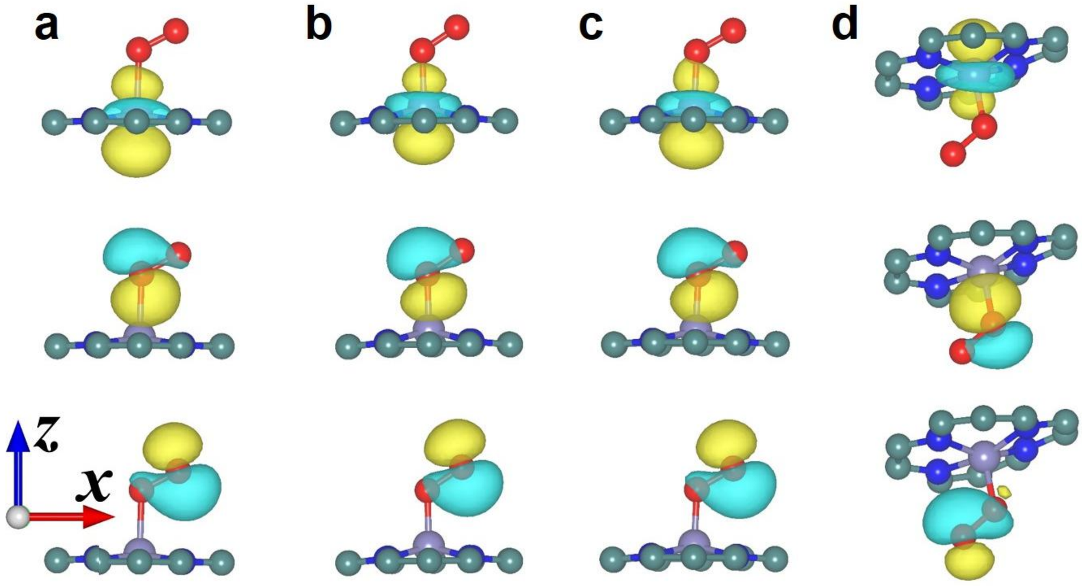

Supplementary Fig. 13 Wannier function. Side view of Fe $3dz^2$ and O_2 2pz Wannier function on **a** O_2@FeN_4, **b** O_2@6, **c** O_2@6a, and **d** O_2@6I.

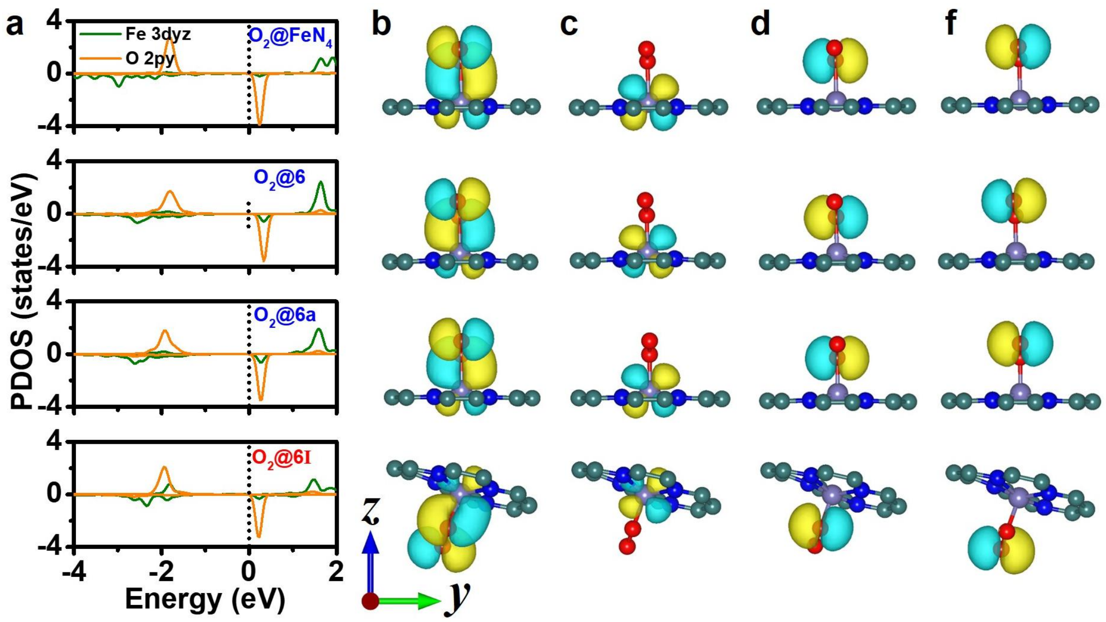

Supplementary Fig. 14 Projected density of states and Wannier function. a PDOS of Fe 3dyz and O_2 2py orbital on O_2@FeN_4, O_2@6, O_2@6a and O_2@6I. **b** Side view of Fe 3dyz and O_2 2py Wannier function. **c** Fe 3dyz Wannier function. **d** O_2 2py Wannier function of proximal O atom. **f** Wannier function of end O atom.

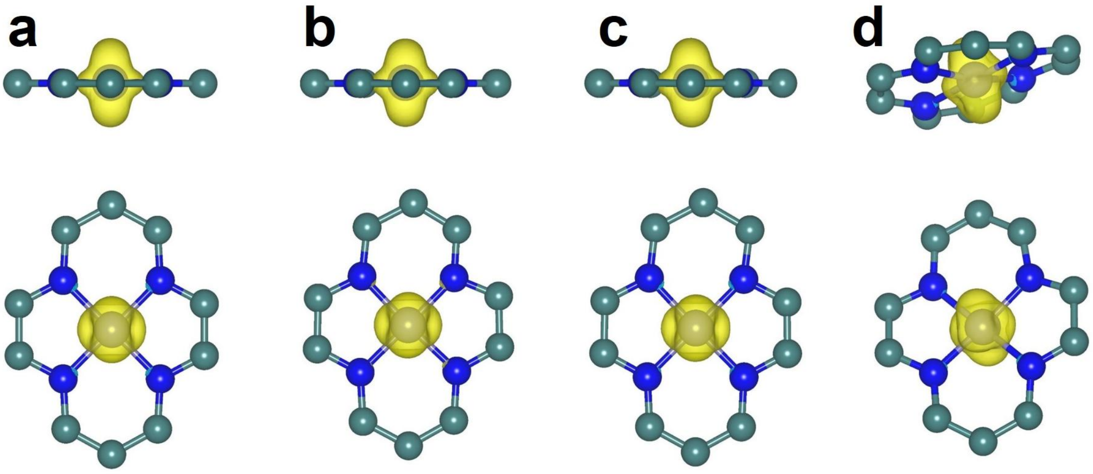

Supplementary Fig. 15 Spin density. Top and side view of spin density of **a** perfect FeN_4, **b** 6, **c** 6a, and **d** 6I.

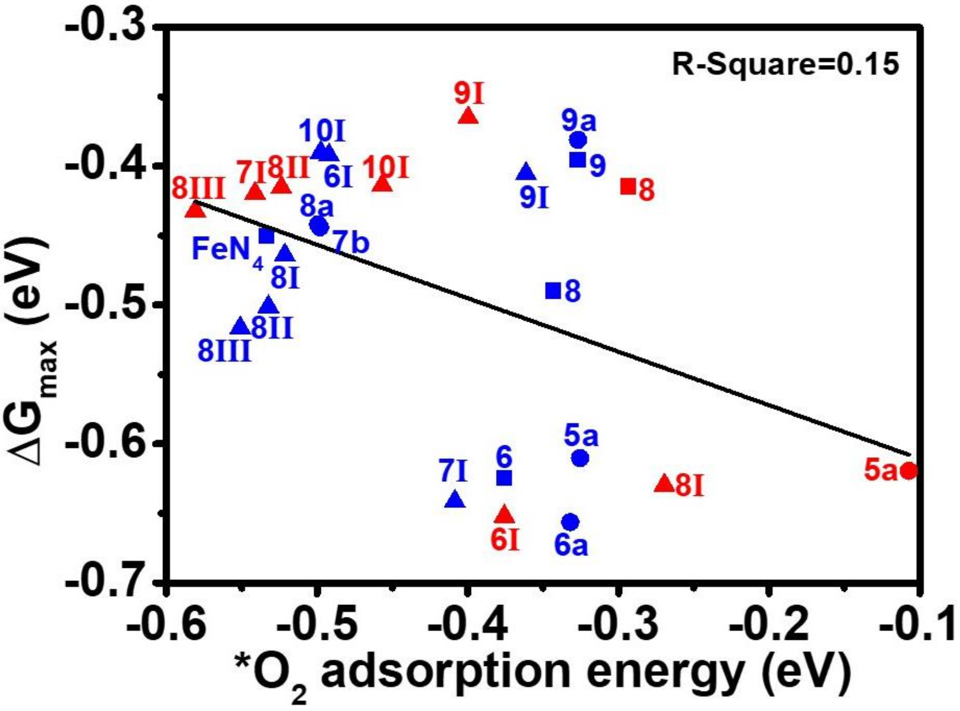

Supplementary Fig. 16 Correlation between the $^*O_2$ adsorption energy and the ORR activity of Fe-N_4 site.

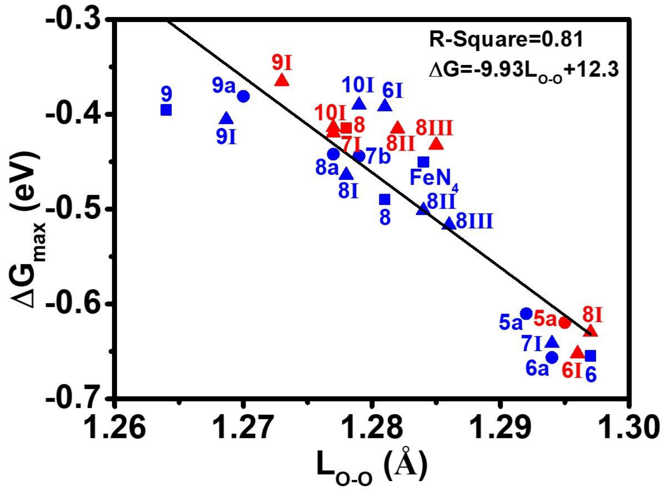

Supplementary Fig. 17 Correlation between the bond length of O–O ($L_{O–O}$) and the ORR activity of Fe-N_4. Blue and red represent upper and lower surface activity, respectively.

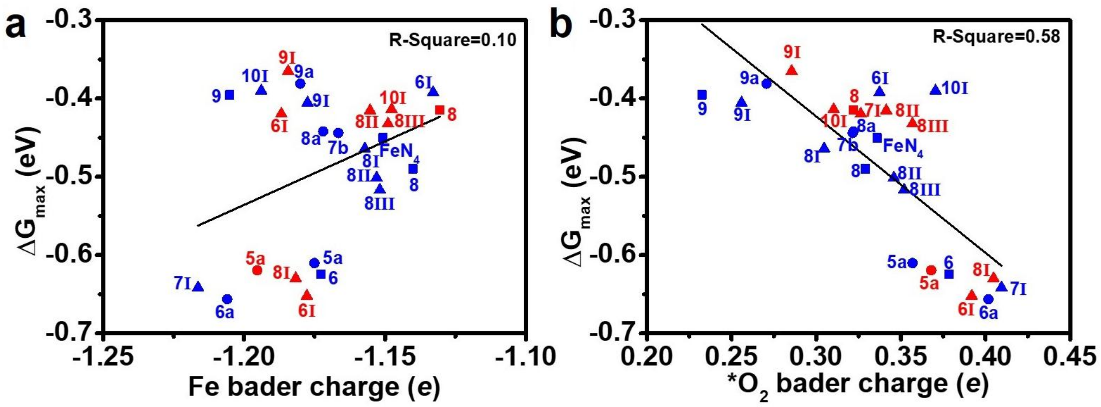

Supplementary Fig. 18 Exploring the descriptors of Fe-N_4 ORR activity. Correlation between the Bader charge of **a** Fe site, **b** *O_2 and the ORR activity of Fe-N_4. Blue and red represent upper and lower surface activity, respectively.

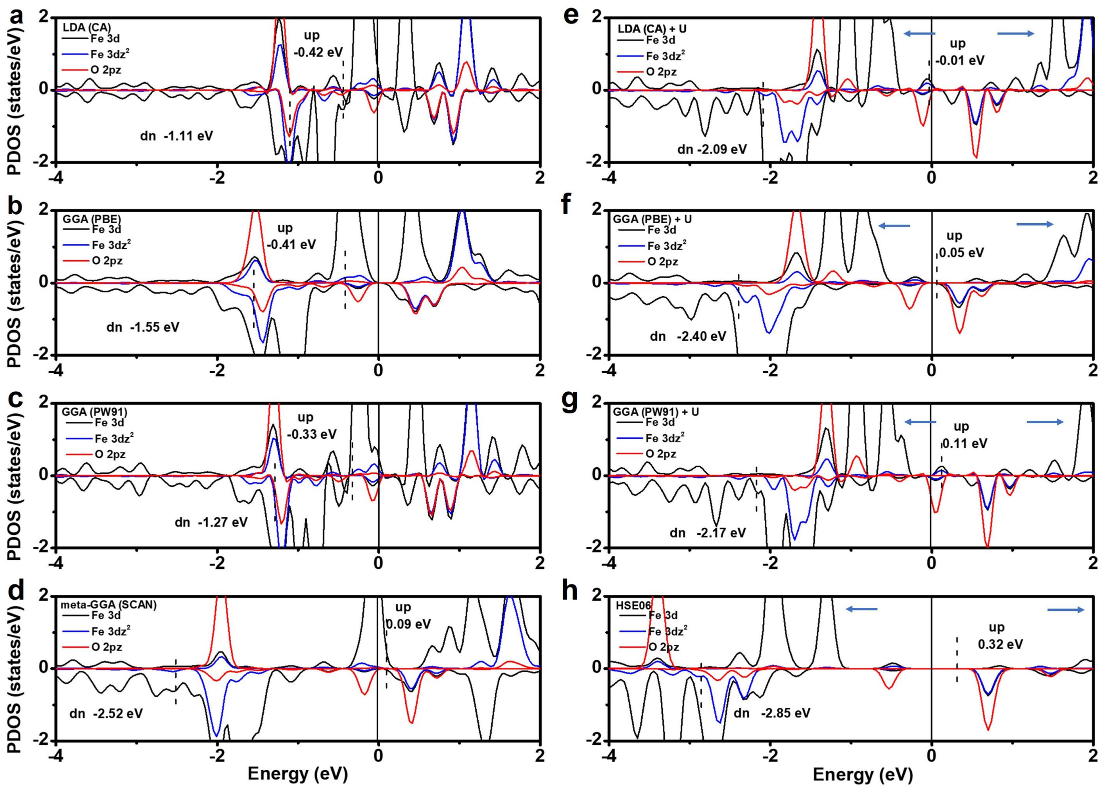

Supplementary Fig. 19 The influence of different exchange-correlation functional. In the theoretical framework of **a** LDA (CA), b GGA (PBE), **c** GGA (PW91), **d** meta-GGA (SCAN), **e** LDA (CA) + U, **f** GGA (PBE) + U, **g** GGA (PW91) + U and **h** HSE06, projected density of states of Fe 3d, $3dz^2$, O_2 2pz orbital in O_2@FeN_4.

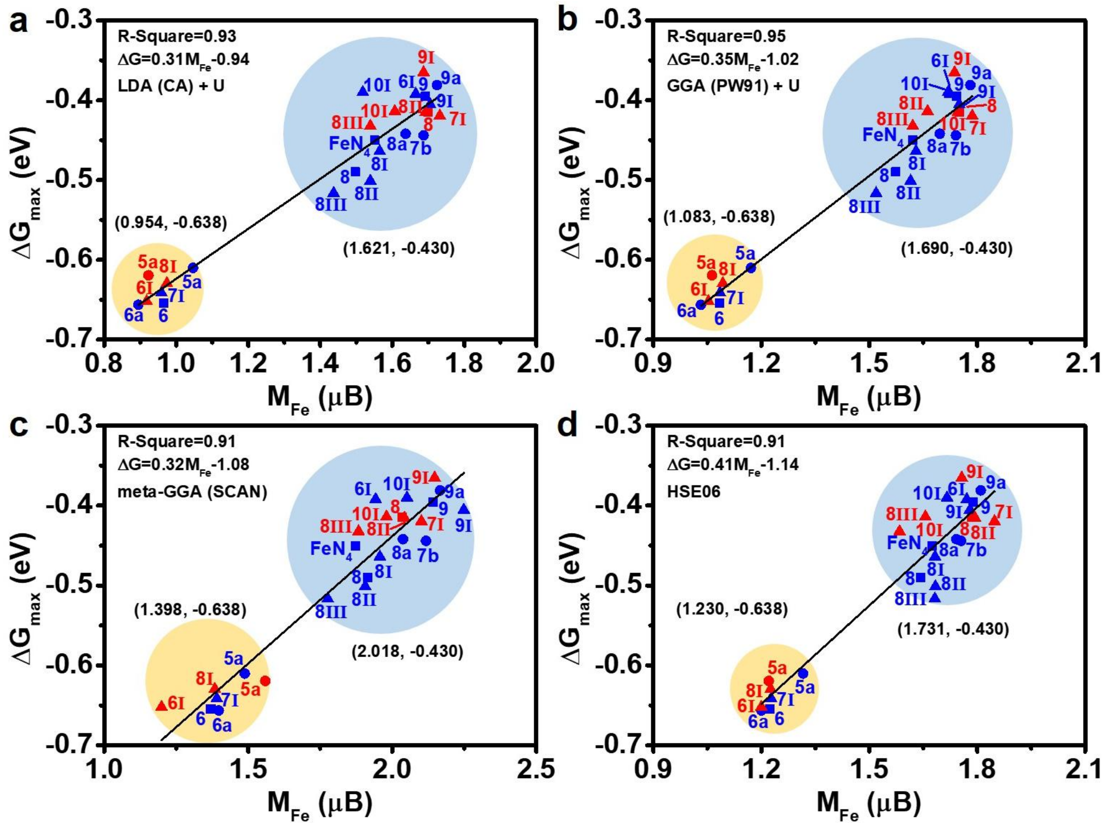

Supplementary Fig. 20 Exploring the descriptors of Fe-N_4 ORR activity. Correlation between M_{Fe} and ΔG in the theoretical framework of **a** LDA (CA) + U, **b** GGA (PW91) + U, **c** meta-GGA (SCAN), and **d** HSE06.

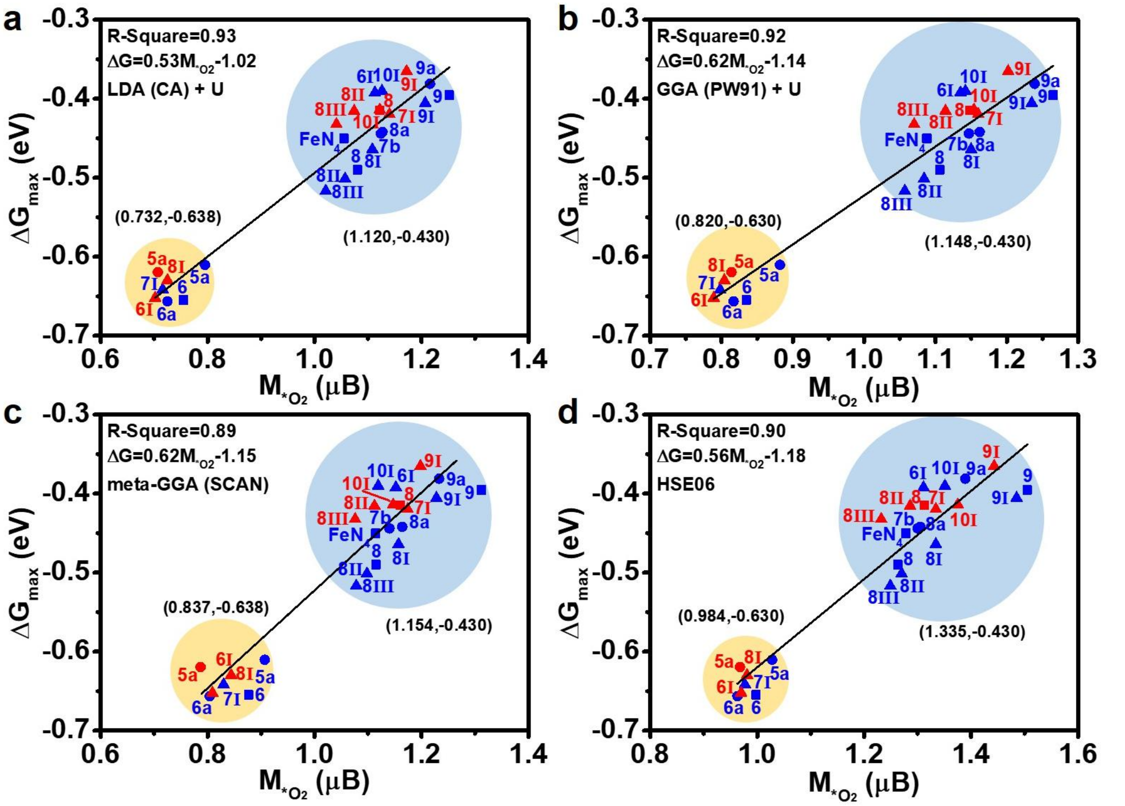

Supplementary Fig. 21 Exploring the descriptors of Fe-N$_4$ ORR activity. Correlation between M_{*O2} and ΔG in the theoretical framework of **a** LDA (CA) + U, **b** GGA (PW91) + U, **c** meta-GGA (SCAN), and **d** HSE06.

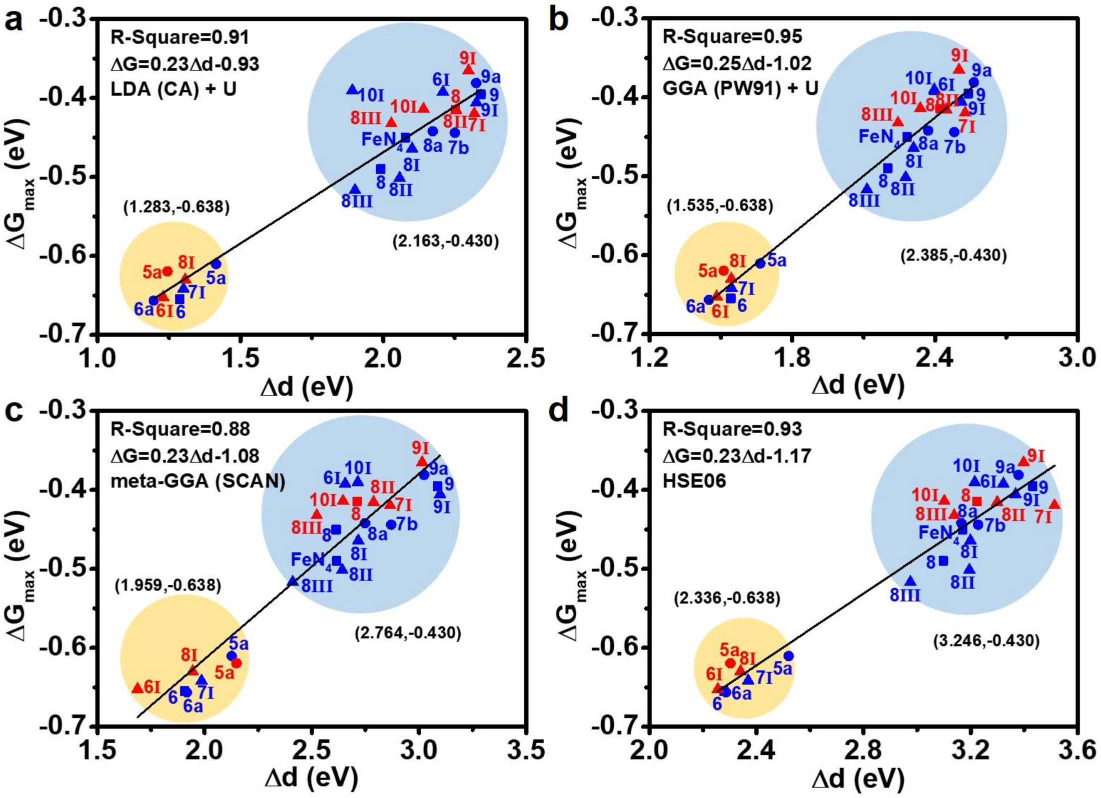

Supplementary Fig. 22 Exploring the descriptors of Fe-N$_4$ ORR activity. Correlation between Δd and ΔG in the theoretical framework of **a** LDA (CA) + U, **b** GGA (PW91) + U, **c** meta-GGA (SCAN), and **d** HSE06.

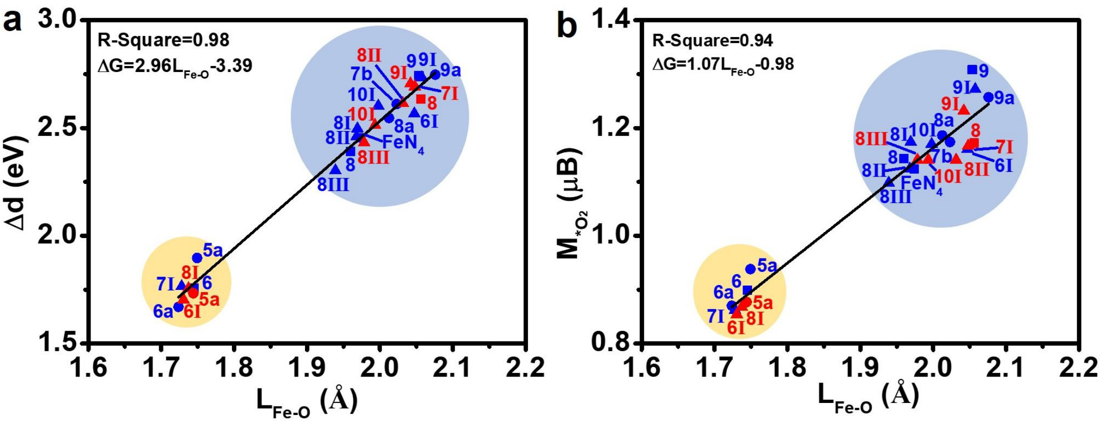

Supplementary Fig. 23 Correlation mechanisms between descriptors. Correlation between **a** d-band center gap of spin state, **b** the magnetic moment of $*O_2$ and the Fe–O bond length ($L_{Fe–O}$). Blue and red represent upper and lower surface data, respectively. Golden and blue circles represent the active and inactive Fe-N_4 site, respectively.

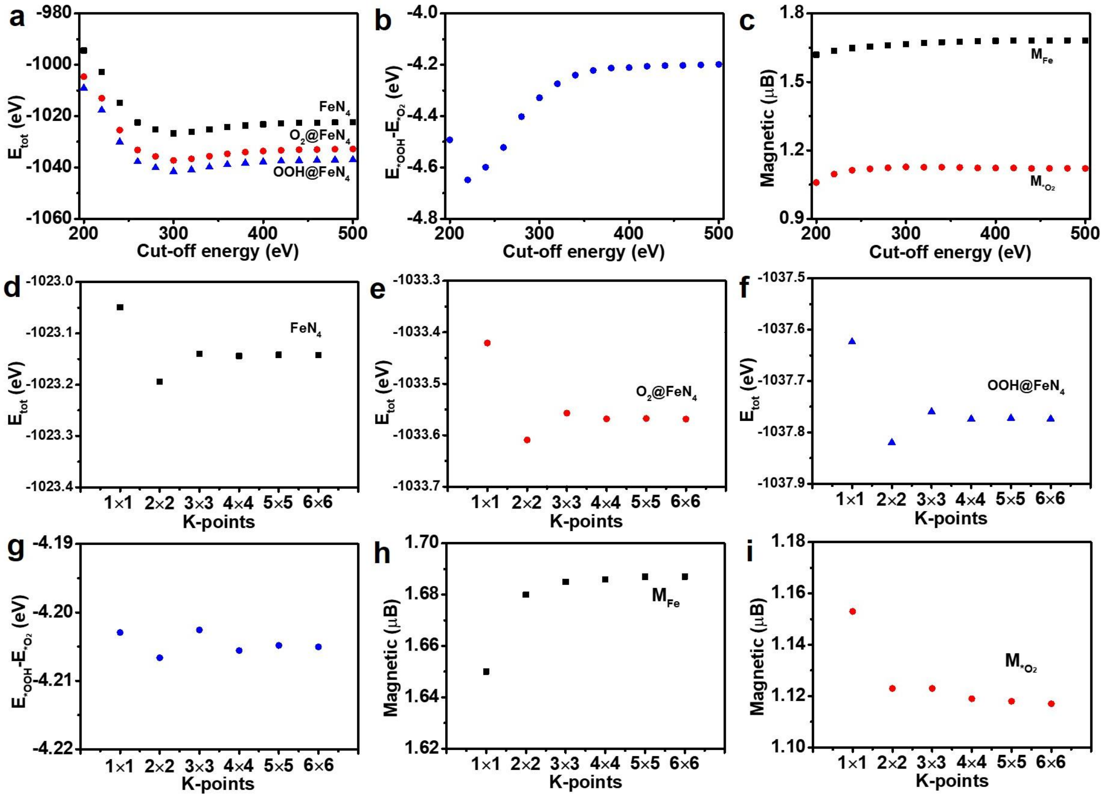

Supplementary Fig. 24 Calculation parameter test. The variation of system energy a, the energy difference of rate-determining step b, and the magnetic moment c with cut-off energy. The total energy of FeN_4 d, $O_2@FeN_4$ e, $OOH@FeN_4$ f, the energy difference of rate-determining step g, the magnetic moment of Fe site h and $*O_2$ i with number of k-points.

Supplementary Table 2. The magnetic moment of Fe sites and $*O_2$, Bader charge of Fe sites and $*O_2$, d-band center gap of spin states (Δd), the bond length of O–O and the bond length of Fe–O after $*O_2$ adsorption.

	M_{Fe}	M_{*O_2}	Charge(Fe)	Charge($*O_2$)	Δd	O-O bonds	Fe-O bonds
FeN_4	1.679 (μB)	1.124 (μB)	-1.151 (e)	0.336 (e)	2.454 (eV)	1.284 (Å)	1.974 (Å)
6	1.191	0.899	-1.173	0.378	1.758	1.297	1.745
8	1.642	1.143	-1.140	0.329	2.391	1.281	1.960
8	1.803	1.172	-1.131	0.322	2.635	1.278	2.056
9	1.805	1.308	-1.205	0.233	2.743	1.264	2.054
5a	1.281	0.938	-1.175	0.357	1.897	1.292	1.749
5a	1.171	0.877	-1.195	0.368	1.733	1.295	1.744
6a	1.144	0.870	-1.206	0.402	1.670	1.294	1.724
7b	1.786	1.174	-1.167	0.322	2.611	1.279	2.023
8a	1.754	1.186	-1.172	0.322	2.546	1.277	2.013
9a	1.830	1.257	-1.180	0.271	2.747	1.270	2.076
6I	1.774	1.161	-1.133	0.337	2.566	1.281	2.047
6I	1.160	0.854	-1.178	0.392	1.703	1.296	1.731
7I	1.190	0.862	-1.216	0.409	1.765	1.294	1.728
7I	1.834	1.167	-1.187	0.326	2.692	1.277	2.048
8I	1.699	1.174	-1.157	0.305	2.497	1.278	1.969
8I	1.196	0.868	-1.182	0.405	1.755	1.297	1.737
8II	1.678	1.124	-1.153	0.346	2.460	1.284	1.968
8II	1.798	1.141	-1.155	0.341	2.615	1.282	2.031
8III	1.592	1.098	-1.152	0.352	2.303	1.286	1.939
8III	1.687	1.141	-1.149	0.357	2.432	1.285	1.978
9I	1.828	1.272	-1.177	0.256	2.735	1.269	2.058
9I	1.793	1.232	-1.184	0.286	2.705	1.273	2.042
10I	1.777	1.170	-1.194	0.370	2.603	1.279	1.998
10I	1.719	1.141	-1.148	0.310	2.514	1.277	1.993

Notes: The blue and red data represent the upper and lower surface of the model, respectively.

Supplementary Table 3. The magnetic moment of Fe sites after $*O_2$ adsorption in the theoretical framework of LDA (CA) + U, GGA (PW91) + U, meta-GGA (SCAN), and HSE06.

	LDA (CA) + U	GGA (PW91) + U	meta-GGA (SCAN)	HSE06
FeN_4	1.551 (μB)	1.621 (μB)	1.842 (μB)	1.675 (μB)
6	0.965	1.084	1.370	1.223
8	1.497	1.574	1.915	1.643
8	1.699	1.750	2.035	1.785
9	1.692	1.743	2.142	1.788
5a	1.047	1.171	1.488	1.316
5a	0.923	1.063	1.559	1.221
6a	0.895	1.032	1.398	1.200
7b	1.687	1.741	2.117	1.755
8a	1.637	1.696	2.037	1.742
9a	1.724	1.781	2.166	1.810
6I	1.665	1.724	1.942	1.771
6I	0.918	1.053	1.199	1.200
7I	0.959	1.085	1.391	1.227
7I	1.732	1.786	2.101	1.848
8I	1.565	1.630	1.957	1.683
8I	0.974	1.093	1.384	1.226
8II	1.539	1.615	1.906	1.684
8II	1.692	1.746	2.042	1.794
8III	1.437	1.519	1.776	1.682
8III	1.539	1.621	1.883	1.584
9I	1.705	1.750	2.248	1.780
9I	1.687	1.737	2.147	1.757
10I	1.518	1.719	2.051	1.716
10I	1.607	1.662	1.980	1.656

Notes: The blue and red data represent the upper and lower surface of the model, respectively.

Supplementary Table 4. The magnetic moment of *O_2 in the theoretical framework of LDA (CA) + U, GGA (PW91) + U, meta-GGA (SCAN), and HSE06.

	LDA (CA) + U	GGA (PW91) + U	meta-GGA (SCAN)	HSE06
FeN_4	1.055 (μB)	1.088 (μB)	1.114 (μB)	1.278 (μB)
6	0.755	0.835	0.877	0.998
8	1.080	1.106	1.115	1.263
8	1.122	1.148	1.160	1.312
9	1.252	1.265	1.312	1.505
5a	0.795	0.882	0.907	1.028
5a	0.707	0.814	0.787	0.968
6a	0.725	0.817	0.804	0.963
7b	1.124	1.147	1.140	1.300
8a	1.127	1.162	1.164	1.305
9a	1.216	1.239	1.233	1.389
6I	1.113	1.135	1.152	1.311
6I	0.702	0.789	0.809	0.970
7I	0.716	0.798	0.830	0.977
7I	1.140	1.160	1.174	1.334
8I	1.108	1.150	1.157	1.334
8I	0.725	0.804	0.844	0.981
8II	1.057	1.084	1.098	1.270
8II	1.074	1.114	1.112	1.286
8III	1.021	1.057	1.078	1.249
8III	1.041	1.070	1.076	1.232
9I	1.207	1.235	1.228	1.485
9I	1.172	1.202	1.198	1.443
10I	1.126	1.142	1.119	1.351
10I	1.122	1.154	1.147	1.375

Notes: The blue and red data represent the upper and lower surface of the model, respectively.

Supplementary Table 5. The d-band center gap of spin states (Δd) after $*O_2$ adsorption in the theoretical framework of LDA (CA) + U, GGA (PW91) + U, meta-GGA (SCAN), and HSE06.

	LDA (CA) + U	GGA (PW91) + U	meta-GGA (SCAN)	HSE06
FeN_4	2.077 (eV)	2.282 (eV)	2.613 (eV)	3.170 (eV)
6	1.288	1.542	1.906	2.278
8	1.991	2.202	2.616	3.098
8	2.252	2.422	2.711	3.223
9	2.341	2.540	3.087	3.432
5a	1.415	1.666	2.126	2.520
5a	1.245	1.512	2.149	2.302
6a	1.197	1.450	1.917	2.286
7b	2.251	2.481	2.871	3.228
8a	2.173	2.371	2.748	3.166
9a	2.325	2.563	3.024	3.379
6I	2.209	2.400	2.656	3.322
6I	1.230	1.483	1.687	2.256
7I	1.301	1.545	1.985	2.369
7I	2.319	2.527	2.865	3.513
8I	2.101	2.310	2.727	3.199
8I	1.307	1.544	1.944	2.340
8II	2.057	2.277	2.642	3.195
8II	2.255	2.447	2.788	3.300
8III	1.901	2.114	2.411	2.975
8III	2.028	2.245	2.523	3.138
9I	2.326	2.511	3.098	3.368
9I	2.298	2.501	3.015	3.398
10I	1.892	2.397	2.715	3.215
10I	2.141	2.339	2.646	3.101

Notes: The blue and red data represent the upper and lower surface of the model, respectively.
